# Nutritional Exposures and Cognitive Impairment in Alcohol Use Disorder: A Structured Narrative Review of Prioritized Human Evidence

**DOI:** 10.3390/nu18213546

**Published:** 2026-10-27

**Authors:** Gabriela Elena Strete, Alexandru Mischie, Lorena Mihaela Grebenisan, Diana Marian, Corina Carmen Radu, Daniela Lucia Muntean, Anca Meda Vasiesiu, Alexandru Andrei Ujlaki-Nagi, Dora-Mihaela Cîmpian, Lorand Ferencz, Ramona Amina Popovici

**Affiliations:** 1 Department of Psychiatry, George Emil Palade University of Medicine, Pharmacy, Science, and Technology of Targu Mures, 540142 Targu Mures, Romania; elena.buicu@umfst.ro (G.E.S.); alexandru.ujlaki@umfst.ro (A.A.U.-N.); 2 Doctoral School, Victor Babes University of Medicine and Pharmacy, 300041 Timisoara, Romania; alexandru.mischie@umft.ro; 3 Department of Dentistry, Faculty of Dentistry, ‘Vasile Goldiș’ Western University of Arad, 94–96 Revoluției Blvd., 310025 Arad, Romania; 4 Department of Forensic Medicine, George Emil Palade University of Medicine, Pharmacy, Science, and Technology of Targu Mures, 38 Gheorghe Marinescu Street, 540139 Targu Mures, Romania; carmen.radu@umfst.ro; 5 Department of Analytical Chemistry, George Emil Palade University of Medicine, Pharmacy, Science, and Technology of Targu Mures, 540142 Targu Mures, Romania; daniela.muntean@umfst.ro; 6 Department of Infectious Disease, George Emil Palade University of Medicine, Pharmacy, Science, and Technology of Targu Mures, Gheorghe Marinescu Street No. 38, 540136 Targu Mures, Romania; anca-meda.vasiesiu@umfst.ro; 7 Department of Bioethics, Deonthology and Medical Communication, George Emil Palade University of Medicine, Pharmacy, Science, and Technology of Targu Mures, 540139 Targu Mures, Romania; dora.cimpian@umfst.ro; 8 Department of Hygiene, George Emil Palade University of Medicine, Pharmacy, Science, and Technology of Targu Mures, 540142 Targu Mures, Romania; lorand.ferencz@umfst.ro; 9 Department Iof-Management and Communication in Dental Medicine, Faculty of Dental Medicine, Victor Babes University of Medicine and Pharmacy, 300041 Timisoara, Romania; ramona.popovici@umft.ro

**Keywords:** alcohol use disorder, cognitive impairment, thiamine, malnutrition, micronutrients, Wernicke–Korsakoff syndrome, neuropsychology, nutritional assessment

## Abstract

Background/Objectives: Cognitive impairment in alcohol use disorder (AUD) may reflect alcohol exposure, withdrawal, nutritional disruption, systemic illness, and social vulnerability. We evaluated prioritized human-adult evidence linking nutritional exposures with objective cognition and examined separately whether AUD severity modifies nutrition–cognition associations and whether nutritional disruption mediates the relationship between alcohol exposure and cognitive outcomes. Methods: This structured narrative review used documented multi-database retrieval, pre-access prioritization, full-text adjudication, evidence appraisal, and qualitative sensitivity scenarios. A targeted vitamin B12 update supplemented the baseline corpus. Results: Thiamine had the broadest and most clinically actionable evidence, but biomarker thresholds, optimal cognitive dosing, and independent treatment effects remained uncertain. Evidence for folate, vitamin B12, niacin outside pellagra, other micronutrients, diet, body mass index, and albumin was sparse, mixed, or nonspecific. The targeted B12 evidence was null or contradictory rather than supportive. Cognitive improvement during detoxification could not be separated from abstinence and bundled care. No included study verified causal mediation by nutrition or a formal nutrition-by-severity interaction. Conclusions: Nutritional assessment and prompt treatment of recognized deficiency remain clinically important, but the prioritized retrieved corpus does not justify nutrient-specific causal claims for typical AUD-related cognitive impairment. Longitudinal studies should align repeated nutrition, alcohol exposure, cognition, and function and prespecify interaction and mediation tests.

## 1. Introduction

Cognitive impairment in alcohol use disorder (AUD) is heterogeneous. Deficits may involve episodic memory, executive function, attention, processing speed, visuospatial ability, and motor performance, and their course ranges from improvement during abstinence to persistent syndromes. Cognitive assessment is therefore relevant beyond overt Wernicke–Korsakoff syndrome [1,2].

Nutrition is one plausible contributor. Chronic alcohol exposure can coexist with restricted intake, impaired absorption, altered nutrient metabolism, liver disease, systemic inflammation, and repeated withdrawal. Thiamine deficiency has the clearest clinical connection through Wernicke encephalopathy and Korsakoff syndrome, while smaller studies address folate, vitamin B12, niacin, antioxidant vitamins, vitamin D, diet, malnutrition, and systemic markers [3].

Competing explanations must be considered before a nutrient-specific account. Direct and indirect alcohol-related neurotoxicity, acute withdrawal, repeated detoxification, hepatic encephalopathy and other hepatic or systemic illness, traumatic brain injury, vascular risk, psychiatric symptoms, other substance use, medication effects, and social deprivation may each influence cognition. Abstinence duration and practice effects can also change test scores. A low nutrient concentration may therefore be a cause, a consequence of illness severity, a treatment-selection marker, or a correlate of broader vulnerability.

The review was organized around two complementary analytical objectives. First, within the prioritized and retrieved human-adult corpus, we examined which nutritional exposures were associated with objectively measured global or domain-specific cognition in AUD. Second, two distinct causal hypotheses were examined: whether AUD severity modifies the association between nutritional exposure and cognition, and whether nutritional disruption mediates the relationship between alcohol exposure and cognitive outcomes. Nutritional exposure denotes a measured biomarker, dietary assessment, diagnosed deficiency syndrome, supplementation regimen, or explicitly defined global nutrition measure. AUD severity denotes a specified measure of current consumption, chronicity, cumulative exposure, withdrawal burden, repeated detoxification, or validated dependence severity. Objective cognition excludes an imaging finding or clinical impression unless accompanied by a standardized cognitive measure. Modification requires a formal interaction or stratified effect estimate; mediation requires an explicit indirect-effect model with defensible temporality. These definitions keep observed associations separate from the testable multi-hit framework used to organize research gaps.

## 2. Methods

### 2.1. Review Design and Analytical Questions

This structured narrative review used documented retrieval, screening, full-text adjudication, extraction, and source-level appraisal. It was not registered as a systematic review and does not claim exhaustive coverage. The analytical questions and operational definitions stated in the Introduction governed the synthesis. Evidence was interpreted within the prioritized retrieved corpus, not as a census of all potentially relevant publications.

### 2.2. Information Sources and Search Strategy

The baseline searches covered PubMed/MEDLINE, Scopus, Web of Science Core Collection, and Europe PMC from inception through 19 August 2026. ScienceDirect served only as a supplementary discovery layer. Search concepts combined AUD or alcohol dependence, nutritional imbalance or nutrient-specific exposures, and cognition or neuropsychological outcomes. Syntax was adapted to each platform. We applied human-adult eligibility through filters where reliable and otherwise through screening. We did not apply an initial publication-date restriction, and the synthesis required full-text English articles. Supplementary File S1 contains the search strings, dates, and amendments used.

Because reviewer feedback identified vitamin B12 as an underdeveloped exposure, a targeted update was run on 3 October 2026 in PubMed and Europe PMC. It combined AUD terms; B12, B-12, cobalamin, and related variants; and broad cognitive terms. Thirty-five deduplicated candidates were screened. One additional eligible full-text study was incorporated [4]. The available search-update information is summarized in Supplementary File S1.

### 2.3. Eligibility, Screening, and Full-Text Adjudication

Eligible sources involved human adults with AUD, alcohol dependence, alcoholism, or a closely related historical construct. Direct clinical studies addressed at least two of the AUD, nutrition, and cognition domains, preferably all three. Human mechanistic and clinical-context sources were retained only for biological plausibility, severe deficiency syndromes, or interpretation and were not treated as direct effect estimates. Animal-only and in vitro studies, adolescent samples, low-risk drinking populations, acute intoxication without relevance to chronic AUD, and liver-disease studies without a meaningful AUD–nutrition–cognition connection were excluded. Case reports were retained only when clinically or mechanistically informative.

The baseline exports contained 939 rows. Removal of 331 exact duplicates left 608 unique records; 221 potentially eligible and 59 uncertain records formed a 280-record acquisition queue. Before access checking, this queue was frozen into P1 direct primary evidence (*n* = 62), P2 direct reviews or guidance (*n* = 13), P3 human mechanistic evidence (*n* = 32), P4 contextual human evidence (*n* = 114), and P5 uncertain records (*n* = 59). Retrieval was attempted for the complete P1–P2 core (*n* = 75). Forty-eight full texts were obtained and assessed, 44 were included, and four were excluded. Twenty-seven core texts were unavailable after attempts (25 without institutional access and two located only in non-English versions). Retrieval was not attempted for the 205 P3–P5 records; their availability and full-text eligibility remain unknown.

**Figure 1:**
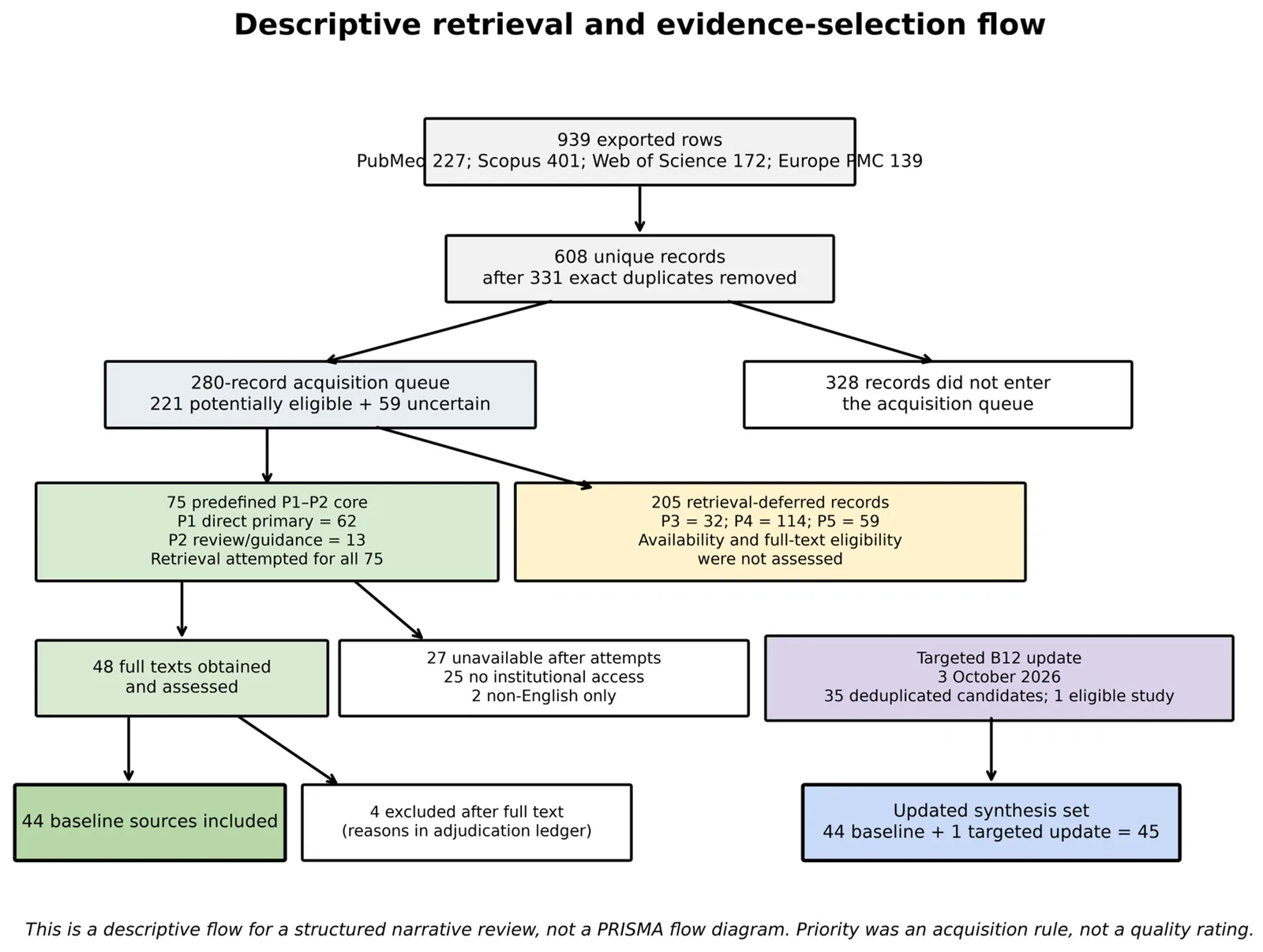
Descriptive retrieval and evidence-selection flow. Priority was an acquisition rule, not a quality rating. The diagram shows the baseline prioritized corpus and the targeted B12 update; it should not be interpreted as a PRISMA flow diagram.

The targeted B12 update added one eligible study to the 44-source baseline, yielding a final synthesis set of 45 sources. The exact search strategies used for the targeted update are provided in Supplementary File S1. We screened and extracted data using an AI-assisted single-reviewer workflow, retaining record-level files for author verification. Extracted information was checked by the authors against the original publications. Independent duplicate screening and independent duplicate data extraction were not performed. Figure 1 illustrates this bounded retrieval process and is not intended as a PRISMA flow diagram.

### 2.4. Data Extraction and Evidence Appraisal

For each included source, extraction captured population and setting, nutritional exposure or mechanism, AUD severity construct, cognitive or neurological outcome, timing, design, finding direction or estimate, confounding and adjustment, limitations, publication status, funding and conflict disclosures when located, and the appropriate evidentiary role. We separated objective cognition from clinical mental status, structural imaging, motor outcomes, and diagnostic codes. AUD diagnosis, current quantity, cumulative exposure, withdrawal burden, and abstinence duration were treated as distinct constructs. Structured extraction was performed once within the AI-assisted workflow and was not independently duplicated. The formatted source-level table is Supplementary Table S1, and the machine-readable traceable evidence map is Supplementary Data S1.

Design levels and source grades were review-specific operational categories rather than a validated risk-of-bias instrument. Level I denoted a systematic review of human studies. Level II denoted randomized controlled interventions. Level III covered non-randomized controlled, comparative, or pre-post intervention evidence, while Level IV covered observational human studies. Level V was reserved for systematic synthesis of descriptive or qualitative evidence and was unused. Level VI denoted case-based or primarily descriptive evidence. Level VII comprised narrative reviews, guidance, commentaries, or tertiary clinical references. Grades considered five dimensions: design execution, directness to the AUD–nutrition–cognition question, measurement and temporality, confounding/precision/attrition, and publication or source verification. Grade A required strong performance across all five dimensions, no major limitations, and no source met this threshold. Grade B allowed cautious central use. Grade C permitted supporting use only with explicit caveats. Grade D restricted a source to context or hypothesis generation. Reviews were not counted as independent confirmation of primary studies they discussed, and case reports were not used to estimate typical effects. We retained the assigned level and grade for positive, null, mixed, and contradictory findings.

### 2.5. AI-Assisted Workflow Statement

ChatGPT (OpenAI, GPT-based language model) was used to assist with bibliographic organization, structured data handling, evidence mapping, citation tracking, and language editing. The authors subjected all 45 sources included in the final synthesis to human verification, systematically cross-checking extracted study characteristics, nutritional exposures, cognitive outcomes, numerical findings, evidence classifications, and supporting references against the original publications. The authors resolved any discrepancies identified during this process by consulting the primary sources. We conducted screening and data extraction within a single-reviewer workflow and did not independently duplicate them. The authors retained full responsibility for study selection, data interpretation, and the manuscript’s scientific accuracy.

### 2.6. Narrative Synthesis

Evidence was grouped by nutrient or nutritional construct, AUD severity and recovery, and measured human mechanisms. Positive, null, mixed, and contradictory results retained their assigned evidentiary level and grade. Mediation, moderation, contextual association, and mechanistic plausibility were coded separately. Four retrospective, record-level sensitivity scenarios were applied during revision: restriction to direct primary human evidence; exclusion of Level VI case-based or descriptive sources; removal of the matched cohort and thiamine systematic review (AUDNC-0177 and AUDNC-0276); and exclusion of the one source that remained a preprint at final verification (AUDNC-0605). These scenarios were not preregistered. Their record-level effects appear in Supplementary Table S2.

## 3. Characteristics of the Evidence Base

The updated synthesis set contained 45 sources: one Level-I systematic review, three Level-II randomized or controlled trials, three Level-III comparative or intervention studies, 21 Level-IV observational studies, eight Level-VI case-based or descriptive sources, and nine Level-VII reviews, guidance documents, commentaries, or tertiary references. Three sources were graded B, 32 C, and ten D; none met the review-specific Grade-A threshold. Twenty-seven sources provided direct evidence, six partial clinical evidence, seven human mechanistic evidence or context, and five guidance or clinical context. These categories describe evidentiary role and do not imply validated risk-of-bias judgments.

The distribution was asymmetric. Thiamine had the largest volume, widest design range, and greatest immediate clinical relevance, but this did not confer high causal certainty. Other nutrients were represented mainly by small cross-sectional studies, pilot interventions, or deficiency-syndrome reports. Few studies measured a defined nutritional exposure, a specified AUD-severity construct, and objective cognition together. No verified study formally established causal mediation by nutrition or moderation by AUD severity (Table 1).

Throughout, “most studied” and “most actionable” refer to research volume, design diversity, and the clinical consequences of missed deficiency. They do not denote a quantified comparative effect or high causal certainty.

## 4. Thiamine and Cognitive Vulnerability

### 4.1. Human Biological and Observational Evidence

Thiamine is biologically linked to brain energy metabolism, yet available human evidence does not support a single diagnostic threshold for cognitive vulnerability. In postmortem tissue from eight patients with alcoholic cirrhosis and six controls, activities of transketolase and selected thiamine-dependent enzymes were reduced in several brain regions despite the absence of overt Wernicke pathology. Cognition was not measured, so the study supports altered cerebral thiamine biology rather than a clinical nutrition–cognition effect [5].

Clinical studies provided convergent but not uniform associations. In a case–control study of 30 adults with AUD and 15 controls, altered thiamine metabolism predicted verbal episodic-memory impairment, whereas broader malnutrition predicted the most severe neuropsychological profile [6]. In a study of 96 adults with chronic alcoholism and 41 controls, low whole-blood thiamine predicted mnemonic deficits, while ataxia was more closely related to non-mnemonic and motor deficits [7]. Adults with comorbid HIV and alcohol dependence who met at least two Caine criteria had the most severe attention/working-memory, delayed-memory, and motor deficits, although HIV, hepatitis C, and polysubstance exposure complicated attribution [8].

Other studies weakened a simple biomarker account. In 19 men assessed early in detoxification, folate was the strongest nutritional correlate of cognitive performance, while thiamine was not associated [9]. A multidomain study of 45 abstinent men found moderate folate-intake deficit but no correlation between nutritional variables and neuropsychological, neurophysiological, or neuroradiological measures [10]. These discrepancies may reflect small samples, different thiamine measures, assessment timing relative to withdrawal, heterogeneous cognitive tests, and restricted biomarker ranges. They also leave open the possibility that peripheral values do not adequately index cerebral availability.

Longer-term observational evidence is suggestive but vulnerable to treatment-selection bias. A nationwide matched cohort compared 5059 thiamine-treated patients with 5059 untreated patients with AUD and found a lower adjusted incidence of dementia in the treated group (hazard ratio approximately 0.55). The association persisted above a cumulative defined-dose threshold, but claims data could not eliminate residual confounding, indication bias, differences in healthcare engagement, or misclassification of nutrition and cognition [11]. The result supports further prevention research. It does not establish that thiamine independently prevents dementia.

### 4.2. Wernicke–Korsakoff Spectrum

Wernicke encephalopathy and Korsakoff syndrome demonstrate that severe thiamine-related brain injury can produce persistent cognitive consequences, but the syndromes should not be used as a direct model for all cognitive impairment in AUD. A focused historical review links alcohol-related thiamine deficiency with heterogeneous cognitive and memory dysfunction while drawing on both human observations and experimental models [12]. Human neuropathology showed modest nucleus-basalis cell loss in both amnesic and non-amnesic alcoholic Wernicke cases, indicating that this lesion alone did not explain profound amnesia [13]. A retrospective cohort of 68 patients with Wernicke–Korsakoff syndrome found that severe infection during the acute phase was common and associated with selected later cognitive scores, further supporting a multi-hit model involving systemic illness and brain vulnerability [14].

Clinical guidance favors rapid recognition and treatment of suspected Wernicke encephalopathy while acknowledging uncertainty about dose and route [15]. This clinical imperative is compatible with uncertainty in comparative cognitive trials: treatment is justified by the consequences of missed deficiency, whereas the research question concerns which regimen produces the best short- and long-term neuropsychological outcome.

### 4.3. Supplementation and Dose

Intervention studies have not defined an optimal cognitive regimen. An early double-blind multidose trial reported better delayed-alternation performance in the 200-mg intramuscular group. Substantial attrition, post-randomization exclusions, short exposure, and a non-monotonic pattern made the finding preliminary [16]. In a prospective detoxification cohort receiving oral thiamine, baseline blood thiamine predicted cognition and both thiamine and MoCA scores improved. However, their association was no longer clear at discharge, when abstinence, withdrawal management, and repeated testing had also occurred [17].

A prospective observational regimen comparison found a marked rise in thiamine pyrophosphate but no regimen difference. The relationship with delayed recall was secondary and uncorrected [18]. Two larger double-blind dose-comparison trials in at-risk and symptomatic participants found no clear cognitive or neurological superiority of higher over lower or intermediate doses during the examined intervals [19]. Because these studies compared active regimens rather than clinically indicated thiamine with no treatment, their null result concerns dose superiority, not whether thiamine is effective when deficiency or Wernicke encephalopathy is present.

A seven-study systematic review likewise found that most studies linked lower thiamine to poorer cognition, sometimes within ranges above conventional deficiency cutoffs, but emphasized substantial heterogeneity in exposures, outcomes, and designs [20]. A linked expert commentary argued that neurocognitive outcomes and possible functional insufficiency deserve attention when thiamine requirements are studied in AUD, but it did not provide an independent effect estimate [21]. The defensible conclusion is therefore narrower than routine claims about dose: thiamine-related vulnerability is clinically important, but circulating thresholds, brain-specific deficiency, optimal route, duration, and cognitive dose–response remain unresolved.

## 5. Non-Thiamine Nutritional Exposures

### 5.1. Folate

Folate evidence was limited and inconsistent. In 19 men assessed early in detoxification, folate was the strongest nutritional correlate of cognitive performance, but extensive correlation testing and the small sample limit stability [9]. A broader study found a moderate folate-intake deficit without a correlation between nutritional variables and neuropsychological, neurophysiological, or neuroradiological measures [10]. MTHFR and vitamin-D-receptor variants did not explain cognitive change during abstinence in 132 inpatients, although these were pathway proxies rather than nutrient measurements [22]. The corpus therefore does not establish a folate-specific cognitive effect in typical AUD.

### 5.2. Vitamin B12

Vitamin B12 was directly assessed in two included observational studies and did not yield a supportive cognition signal. In a retrospective detoxification cohort of 52 patients, the univariate association between B12 and MoCA was null (β = 5.6 × 10^−4^, *p* = 0.84), whereas ascorbic acid showed an adjusted association [23]. In the targeted-update study of 59 alcohol-dependent adults and 24 controls, B12 concentrations were higher in the alcohol-dependent group and tracked liver dysfunction and computed-tomography atrophy; no measured variable was independently related to MMSE [4]. These results argue against treating low circulating B12 as an established explanation for cognitive impairment in AUD. They do not exclude clinically diagnosed B12 deficiency in individual patients or effects that require different biomarkers, cognitive instruments, or longitudinal timing.

A further historical study measured B12 in alcoholism but was not available in full text and did not provide a verified B12-specific cognitive model; therefore, we did not use it as outcome evidence. The targeted search found two multivitamin case reports that could not isolate B12 and were retained only as screening context. This distinction prevents a general deficiency narrative from being mistaken for B12-specific evidence.

### 5.3. Niacin and Pellagra

Niacin evidence came mainly from pellagra reports. Patients presented with altered mental status or cognitive dysfunction and improved after nicotinamide or niacin-containing treatment [24,25]. In one case, MoCA rose from 20 to 29 during a five-day admission that also included other vitamins, detoxification, and medical care [26]. These cases keep pellagra in the differential diagnosis for severe nutritional compromise, but they cannot estimate prevalence, identify subclinical effects, or isolate niacin from concurrent treatment.

### 5.4. Vitamins A, C, D, and E

Antioxidant-vitamin studies produced outcome-specific results. Vitamins A and E were lower in a withdrawal cohort and related to selected computed-tomography indices, but neither vitamin nor B12 was associated with MMSE [27]. A case combining severe deficiencies of vitamins A, D, and E with objective cognitive impairment retained substantial disability after biochemical correction, and the contribution of each deficiency could not be separated [28].

Ascorbic acid provided a preliminary positive signal: 28.1% of a detoxification cohort was deficient, and log ascorbic acid independently predicted MoCA (β = 1.18, *p* = 0.037) [23]. Missing cognitive data, clinical selection, and cross-sectional timing limit inference. Vitamin D was not significantly different between cognitively impaired and unimpaired alcohol-dependent participants in a study where all groups were deficient [29].

### 5.5. Antioxidant Interventions

A preliminary randomized placebo-controlled trial of N-acetylcysteine in 23 adults with moderate-to-severe AUD found no significant group differences in neurometabolites or cognitive scores after approximately 19 days [30]. A very small two-week pilot reported MMSE improvement only with polydatin, but allocation, abstinence, and concurrent care limited attribution [31]. These studies neither establish nor exclude benefit; they show the need for adequately powered trials with prespecified cognitive outcomes.

## 6. Global Nutritional Status, Diet, and Systemic Markers

Global nutritional vulnerability may be more informative than any single nutrient in some patients, but measurement remains problematic. In the 30-patient case–control study, a composite malnutrition classification predicted the most severe cognitive profile [6]. Among patients with compensated alcoholic cirrhosis, lower BMI and dietary pattern were associated with selected K-MMSE domains, although liver disease and the cross-sectional design precluded a nutritional causal interpretation [32].

BMI did not behave as a general predictor. In 86 patients tested during abstinence, admission BMI was associated only with two speed or visuomotor outcomes and did not predict overall neurocognitive recovery between weeks two and six [33]. BMI cannot distinguish lean mass, adiposity, edema, acute intake, micronutrient deficiency, or prior weight loss. A null BMI finding therefore does not show that nutrition is irrelevant. It shows that this proxy is too crude for a general causal claim.

The OLA mixed-methods study highlighted the complexity of older adults. Among 30 hospital patients aged over 64 and identified through alcohol screening, 53% screened positive for cognitive impairment and median intake for four of five macronutrients was below recommendations. The study did not estimate a nutrition–cognition association, and attrition reached 46.7%, but it linked nutritional vulnerability with depression, social isolation, mortality, and difficulty retaining participants in longitudinal research [34].

Serum albumin illustrates the interpretive challenge of systemic markers. In a published multisample analysis of 160 adults with AUD, 142 with HIV, and 102 controls, lower albumin contributed independent variance to periventricular white-matter hyperintensity burden and visual and verbal learning or memory after adjustment. Albumin also related to liver and hematological indices, making it a marker of systemic vulnerability rather than a nutrient-specific exposure [35]. This finding supports an integrated nutritional, hepatic, inflammatory, and vascular assessment, but not albumin replacement as a cognitive intervention.

## 7. AUD Severity, Abstinence, and Limits of Causal Attribution

### 7.1. Severity, Moderation, and Mediation

The central severity hypothesis was weakly addressed. Some studies measured consumption history, nutritional markers, and cognition together [6,7,8], yet none provided a persuasive prespecified nutrition-by-severity interaction or causal mediation estimate. Adjustment for alcohol history is not evidence that severity changes a nutritional effect. Diagnostic status, current quantity, chronicity, cumulative exposure, withdrawal burden, and repeated detoxification also represent different estimands and should not be collapsed into a single severity label.

Adjacent biomarkers do not close this gap. Plasma proBDNF related to severity and cognition, but nutrition was not measured [36]. Trimethylamine N-oxide and indoxyl sulfate were unrelated to severity, withdrawal, or cognition in a small study [37]. These findings are contextual fragments, not evidence for nutritional mediation. Until studies align repeated measurements and explicitly test indirect and interaction effects, severity moderation and nutritional mediation remain hypotheses.

### 7.2. Abstinence, Supplementation, and Recovery

Cognitive improvement during abstinence complicates treatment attribution. A five-year case showed marked neuropsychological and FDG-PET recovery without thiamine deficiency [38]. In a thiamine-supplemented detoxification cohort, blood thiamine and cognition improved together, but their baseline association was no longer clear at discharge [17]. A Romanian inpatient cohort showed an MMSE increase during two weeks of abstinence, B1/B6 vitamins, Cerebrolysin, and usual care [39]. Withdrawal resolution, repeat testing, regression to the mean, and co-interventions prevent nutrient-specific attribution.

The distinction is clinical as well as causal. Recognized deficiency and suspected Wernicke encephalopathy require prompt treatment, while evidence remains insufficient to quantify the independent cognitive benefit of a specific nutrient regimen in typical AUD. Pellagra cases improved during multicomponent care, whereas a patient with multiple deficiencies retained substantial disability after biochemical correction [26,28]. Recovery therefore cannot be used retrospectively to prove which exposure caused the initial impairment.

## 8. Measured Human Mechanisms and a Testable Framework

### 8.1. Measured Links

Human findings support fragments of a mechanism, not an end-to-end causal chain. Thiamine-dependent enzyme activities were reduced in postmortem alcoholic-cirrhosis brain tissue without classic Wernicke pathology, but cognition was not measured [5]. Nucleus-basalis cell loss occurred in amnesic and non-amnesic alcoholic Wernicke cases, weakening a single-lesion explanation for persistent amnesia [13]. Structural MRI correlates of AUD-sensitive cognitive screening showed distributed substrates without demonstrating nutritional mediation [40]. Albumin, liver-related indices, proBDNF, vitamins, and microbial metabolites showed additional associations or null results [27,35,36,37]. Their evidentiary roles are described in the corresponding sections and summarized in Supplementary Table S1.

### 8.2. Contextual Fragments and Untested Pathways

Withdrawal, abstinence, hepatic or systemic illness, infection, and social vulnerability were measured in parts of the corpus, but rarely alongside repeated nutrient and cognitive measures [14,17,34,36,39]. Hypotheses involving blood–brain-barrier disruption, cerebral iron, or a complete gut–liver–brain sequence arose from reviews and were not tested end-to-end in the adult synthesis set [41,42]. They are research gaps rather than observed mechanisms. Related evidence outside the AUD-specific literature also supports a broader diet–microbiome–brain framework linking nutritional exposures with neurobehavioral and mental-health outcomes through microbial, inflammatory, and metabolic pathways [43]. However, these findings remain contextual and should not be extrapolated directly to AUD-related cognitive impairment.

Marchiafava–Bignami disease illustrates convergence at the severe end of the spectrum. A clinical reference describes a rare callosal disorder associated with chronic alcohol use or malnutrition [44]. A preprint case lacked standardized cognitive testing and a measured thiamine value [45]. In a retrospective series of 21 men, longer drinking history and lower thiamine supplementation were associated with poorer one-year prognosis, but treatment selection and clinical outcome ascertainment limit causal inference [46]. These sources should not be used as proxies for typical AUD-related cognitive impairment.

### 8.3. Proposed Tests

**Figure 2:**
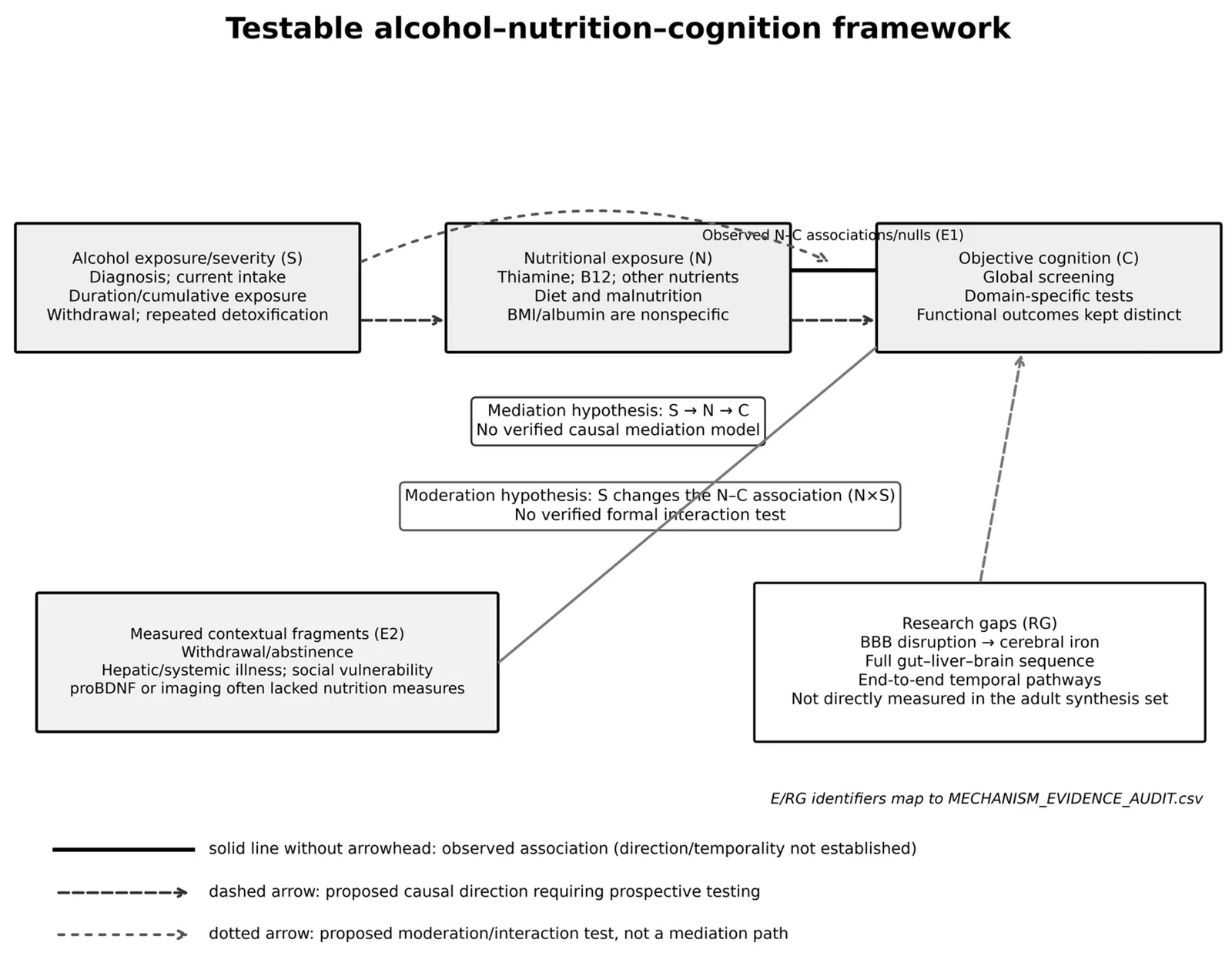
Testable alcohol–nutrition–cognition framework. Solid line without an arrowhead: observed association or null result. Dashed arrows: proposed causal direction requiring prospective testing. Dotted arrow: proposed moderation/interaction test, distinct from mediation. Evidence identifiers refer to observed nutrition–cognition associations (E1; [6,7,9,10,23]) and contextual mechanistic findings (E2; [5,13,14,35,36,37]). The research-gap identifier (RG1; [41,42]) denotes proposed mechanisms that remain insufficiently validated in human studies.

Figure 2 separates three relationships. The solid, arrowless nutrition–cognition line represents observed associations and null results for which direction and temporality are unresolved. Dashed arrows represent the proposed causal mediation sequence from alcohol exposure through nutrition to cognition. The dotted path represents a distinct moderation hypothesis in which AUD severity changes the nutrition–cognition association. Neither the mediation nor interaction model was verified in the updated synthesis set.

The model is testable. Longitudinal studies should define the alcohol-exposure estimand, obtain repeated nutrition and domain-specific cognitive measures after stabilization, and retain functional outcomes as a separate endpoint. Mediation analyses require exposure to precede change in the proposed mediator and outcome, with confounders stated in a causal diagram. Moderation analyses require prespecified interaction terms, adequate power, and stratum-specific estimates. Trials should not withhold clinically indicated thiamine but can compare accepted routes, doses, and durations while standardizing co-interventions. The key methodological requirements for testing the proposed framework, including temporal alignment, nutritional assessment, AUD severity characterization, and causal inference, are summarized in Table 2.

## 9. Discussion

### 9.1. Three Principal Findings

First, thiamine was the most studied and clinically actionable nutritional exposure, not the most causally certain. Biomarker and observational studies, neuropathology, a matched cohort, and active-regimen comparisons support clinical salience, especially around Wernicke–Korsakoff syndromes [5,6,7,8,11,13,14,15,19,20]. They do not define a universal biomarker threshold, prove a monotonic cognitive dose–response, or isolate dementia prevention from treatment selection.

Second, evidence for other nutritional exposures was sparse and exposure-specific. Folate findings conflicted [9,10,22]. B12 showed null or contradictory cognition findings in the two eligible studies [4,23]. Niacin evidence was confined mainly to pellagra cases under bundled care [24,25,26]. Selected associations for ascorbic acid, global malnutrition, diet, and albumin require replication, while BMI, vitamin D, N-acetylcysteine, and microbial-metabolite studies supplied useful null or limited findings [23,29,30,31,32,33,34,35,37].

Third, the proposed role of AUD severity was not established. No verified study demonstrated a formal nutrition-by-severity interaction or a temporally defensible nutritional mediation pathway. Cognitive change during detoxification and abstinence occurred amid withdrawal resolution, repeated testing, supplements, and other care [17,38,39]. The framework in Figure 2 therefore organizes explicit tests; it is not a validated mechanism. Under sensitivity scenario SENS-1, the multi-hit framework was classified as attenuated but not reversed, reflecting reduced evidentiary support after excluding contextual and mechanistic sources. This finding reinforces its interpretation as a hypothesis-generating conceptual framework rather than a validated causal model.

### 9.2. Clinical Interpretation

Clinical practice should distinguish prevention of irreversible deficiency-related harm from claims of cognitive optimization. Suspected Wernicke encephalopathy warrants prompt management despite uncertainty about the best comparative regimen [1,15]. Broader assessment should start with recent intake, weight trajectory, malnutrition risk, physical signs, comorbidities, and social barriers, then proceed to targeted testing when a specific deficiency or management decision is plausible. BMI and albumin are nonspecific and should not be treated as stand-alone nutrient diagnoses.

The evidence does not support universal high-dose nutrient regimens to improve cognition in all adults with AUD. Pellagra and clinically diagnosed B12 deficiency remain treatable conditions, but their recognition should not be generalized into unsupported causal explanations for typical cognitive impairment. Cognition and function should be reassessed after withdrawal has resolved and care has stabilized. Domain-based testing is preferable when impairment affects treatment engagement, capacity, rehabilitation, or discharge planning.

### 9.3. Research Priorities

Prospective cohorts should recruit across defined alcohol-exposure strata and align repeated dietary, biomarker, liver, inflammatory, cognitive, and functional assessments. Current intake, chronicity, cumulative exposure, withdrawal burden, and treatment history should be modeled separately. Prespecified causal diagrams should identify confounders and clarify which estimand is being tested.

Clinically ethical trials can compare accepted routes, doses, or durations, stratify by baseline deficiency, standardize co-interventions, and measure medium- and long-term domain-specific cognition. The most important reporting improvement is not another broad mechanistic narrative but publication of interaction, indirect-effect, and null estimates with uncertainty.

### 9.4. Strengths and Limitations

Strengths include multi-database retrieval, reconstruction of a frozen pre-access priority rule, transparent separation of direct and contextual evidence, full-text-based extraction, preservation of null and contradictory results, a targeted B12 update, and record-level sensitivity scenarios. The 205 deferred P3–P5 records are explicitly acknowledged as not having undergone full-text eligibility assessment.

The limitations are substantial. This narrative review is not exhaustive. Retrieval was attempted for only the 75-record P1–P2 core; 27 of those texts remained unavailable, and 205 P3–P5 records were deferred without access or full-text eligibility assessment. The 45-source synthesis is therefore bounded by prioritization and access. This prioritization strategy introduces a potential source-selection bias, as the 205 records not subjected to full-text assessment may contain relevant evidence that was not captured in the synthesis. Although these records were primarily classified as mechanistic, contextual, or uncertain based on bibliographic information, we could not verify their eligibility or findings. Consequently, we may have missed potentially supportive, contradictory, or null findings, particularly for less extensively studied nutritional exposures and mechanistic pathways. This limitation may influence the relative weight assigned to individual evidence domains and restricts the generalizability of the conclusions beyond the prioritized and verified corpus.

Screening and extraction were not independently duplicated. Most direct studies were observational, small, male-predominant, and heterogeneous. Severe-syndrome cases cannot estimate typical effects. Nutritional biomarkers were often peripheral and measured once, cognition was frequently global rather than domain-specific, and temporal alignment was weak. One included source remained a preprint at final verification. The sensitivity scenarios were retrospective and qualitative, not preregistered or meta-analytic. No scenario reversed the central conclusions, but missing and deferred studies could change the balance or historical depth of individual themes.

## 10. Conclusions

Within the prioritized retrieved corpus and targeted B12 update, thiamine was the most studied and clinically actionable nutritional exposure, while evidence for other nutrients was sparse, mixed, or nonspecific. The literature did not establish nutritional mediation or modification by AUD severity, and recovery during detoxification could not be attributed to a single nutrient. Clinical care should continue to identify and treat recognized deficiency promptly, while research should test temporality, interaction, and mediation with aligned repeated measures rather than infer a causal multi-hit pathway from disconnected associations.

## Figures and Tables

**Table 1:** Summary of evidence domains and inferential limits.

Domain	Main Human Evidence	Direction	Bounded Interpretation
Thiamine	Biomarkers, cohort, systematic review, dose comparisons, neuropathology	Mostly supportive associations; mixed intervention evidence	Clinically salient; thresholds and cognitive dose–response uncertain
Folate	Small observational studies and pathway proxies	Mixed	Sparse and not independently replicated
Vitamin B12	Observational biomarker studies	Null or contradictory for cognition	No evidence of a B12-specific cognitive effect in AUD
Niacin/pellagra	Case reports	Clinical improvement during bundled treatment	Relevant to overt deficiency; not generalizable to typical AUD
Other micronutrients/antioxidants	Cross-sectional studies and small pilots	Selected positive and several null findings	Preliminary and nutrient-specific
Global nutrition/diet	Malnutrition profiles, BMI, diet, albumin	Selected associations plus nulls	Measures are nonspecific and noncausal
AUD severity/recovery	Covariate data, abstinence cohorts, bundled care	Recovery occurs; formal interaction absent	Treatment and abstinence confound attribution

**Table 2:** Priority design features for testing the framework.

Problem	Required Design Feature	Minimum Reporting Requirement
Unclear temporality	Repeated assessment after medical stabilization	Last drink, withdrawal resolution, abstinence duration, repeated nutrition/cognition
Nutrition measurement error	Diet, malnutrition screen, body composition, repeated biomarkers	Assay, threshold, supplements, inflammation, liver function
Severity construct mixing	Separate current intake, chronicity, cumulative exposure, withdrawal	Instrument, recall window, distribution, prespecified estimand
Cognitive heterogeneity	Domain-based battery plus separate functional outcomes	Domain scores, impairment threshold, practice-effect strategy, missingness
Interaction/mediation gap	Prespecified causal diagram and adequately powered models	Estimates with uncertainty, assumptions, multiplicity, null results
Treatment attribution	Accepted-regimen trials stratified by baseline deficiency	Co-interventions, adherence, harms, medium- and long-term cognition

## Data Availability

We did not create new participant-level data. We provide the executed search strategies (File S1), source-level study table (Table S1), traceable evidence map (Data S1), qualitative robustness scenarios (Table S2), and core full-text access profile (Data S2). Database exports, screening decisions, extraction files, and locally stored full texts remain subject to database licenses and copyright and are available from the corresponding author where sharing is permitted.

## Supplementary Materials

The following supporting information can be downloaded at: https://www.mdpi.com/article/10.3390/nu18213546/s1, File S1: Executed search strategies and amendments; Table S1: Study characteristics and review-specific appraisal; Data S1: Traceable evidence map; Table S2: Qualitative sensitivity scenarios and conclusion impact; Data S2: Core full-text access profile.
