# Supplementary material for: Nutritional Exposures and Cognitive Impairment in Alcohol Use Disorder: A Structured Narrative Review of Prioritized Human Evidence
Source: Nutrients. 2026 Oct 27;18(21):3546. doi: 10.3390/nu18213546 (PMC13765479; doi:10.3390/nu18213546)
Supplement: Supplementary file 1 [file nutrients-18-03546-s001.zip › Supplementary File S1.pdf]

# Supplementary File S1

**Executed Search Strategies, Dates, and Export Provenance**

Review: Nutritional Exposures and Cognitive Impairment in Alcohol Use Disorder: A Structured Narrative Review of Prioritized Human Evidence

## Scope and Harmonization

The formal retrieval used one shared conceptual Boolean: AUD AND nutrition or prioritized nutrients AND objective cognition. Database-specific changes were limited to field syntax and controlled vocabulary. PubMed used MeSH-based human-adult restrictions. Scopus, Web of Science, and Europe PMC applied strict human-adult eligibility during screening because requiring population words in title or abstract fields produced unacceptable false-negative risk.

## Executed Database Searches

| Database | Version | Date | Records | Export | SHA-256 |
|---|---|---|---|---|---|
| PubMed/MEDLINE | v3 | 19 August 2026 | 227 | searches/pubmed/PUBMED_MASTER_v3.xml | A27D9E72BDCF56CBA7A4C80B3F43560E9AE3EC7CE2D91D063F2290962E4BC05E |
| Scopus | v4.1 | 19 August 2026 | 401 | searches/scopus/raw_exports_v4_1/SCOPUS_MASTER_v4_1.csv | 65201580495ABE9E750DD2ADEA4A887987D7D294FFD19EB744EFEC959A5B555B |
| Web of Science Core Collection | v4.1 | 19 August 2026 | 172 | searches/wos/raw_exports_v4_1/WOS_MASTER_v4_1.txt | 3300F11324E65ABE071906B583195D1849D8F4CA17D6D9AE13AF3756A2885B21 |
| Europe PMC | v4.1 | 19 August 2026 | 139 | searches/europe_pmc/raw_exports_v4_1/EUROPE_PMC_MASTER_v4_1.ris | 6E93A76285C67564595BA44446540DDED040B3B97E827D145C4DE2F1E09D7069 |

The four formal exports contained 939 source rows. Exact identifier and normalized-title deduplication merged 331 duplicate rows, leaving 608 unique records for title/abstract screening. Of these, 221 were considered potentially eligible and 59 remained uncertain at title/abstract screening, forming a 280-record full-text acquisition queue. Seventy-five records comprised the prioritized core full-text set. Forty-eight were locally available and adjudicated, yielding 44 included and four excluded full texts; 27 core full texts remained unavailable locally.

The remaining 205 records belonged to the P3–P5 mechanistic, contextual, or uncertain categories. We did not attempt full-text retrieval for these records, which remained deferred in the acquisition queue; consequently, we did not assess their full-text availability or eligibility. None contributed to the baseline 44-source synthesis. The baseline included corpus therefore consisted entirely of records from the predefined P1/P2 core evidence set. A subsequent targeted vitamin B12 update added one eligible source, yielding a final 45-source synthesis; this update was conducted separately from the baseline exports and counts.

ScienceDirect was used only as a supplementary publisher-interface discovery layer. It was not included in the 939 formal export rows or the cross-database deduplication count.

## Search Amendments

1. The exploratory multi-family architecture was replaced before formal screening by one master Boolean suitable for a narrative review.
2. PubMed retained controlled Humans and adult-age MeSH restrictions.
3. The free-text human/adult proxy was removed from Scopus, Web of Science, and Europe PMC after it reduced Europe PMC retrieval to six records and exceeded the platform's 4,000-character export threshold.
4. Human-adult eligibility remained strict and was enforced during screening in databases without reliable controlled population filters.
5. Web of Science and Europe PMC were added before final deduplication while preserving the same three topical concepts.
6. Earlier query families and pilot versions were retained only as audit artifacts and did not define the final screened corpus.

## Exact Executed Strings

### PubMed/MEDLINE (v3)

```
(
  "Alcohol-Related Disorders"[Mesh]
  OR "Alcohol-Induced Disorders, Nervous System"[Mesh]
  OR "Alcoholic Korsakoff Syndrome"[Mesh]
  OR "Alcoholism"[Mesh]
  OR "alcohol use disorder"[tiab]
  OR "alcohol use disorders"[tiab]
  OR "alcohol-related disorder"[tiab]
  OR "alcohol-related disorders"[tiab]
  OR "alcohol dependence"[tiab]
  OR "alcohol dependent"[tiab]
  OR "alcohol abuse"[tiab]
  OR "alcohol misuse"[tiab]
  OR "alcohol addiction"[tiab]
  OR alcoholism[tiab]
  OR "chronic alcohol use"[tiab]
  OR "chronic alcohol consumption"[tiab]
  OR "heavy alcohol use"[tiab]
  OR "heavy drinking"[tiab]
  OR "alcohol-related brain damage"[tiab]
  OR "alcohol related brain damage"[tiab]
  OR ARBD[tiab]
)
AND
((
  "Malnutrition"[Mesh]
  OR "Nutritional Status"[Mesh]
  OR "Nutrition Disorders"[Mesh]
  OR malnutrition[tiab]
  OR malnourish*[tiab]
  OR undernutrition[tiab]
  OR "nutritional deficiency"[tiab]
  OR "nutritional deficiencies"[tiab]
  OR "nutrient deficiency"[tiab]
  OR "nutrient deficiencies"[tiab]
  OR "nutritional imbalance"[tiab]
  OR "nutritional imbalances"[tiab]
  OR "nutritional status"[tiab]
  OR "nutrient status"[tiab]
  OR "dietary intake"[tiab]
  OR "dietary inadequacy"[tiab]
  OR "nutritional inadequacy"[tiab]
  OR "poor nutrition"[tiab]
  OR micronutrient*[tiab]
  OR "protein-energy malnutrition"[tiab]
```

```
  OR "protein energy malnutrition"[tiab]
) OR (
  thiamine[tiab]
  OR thiamin[tiab]
  OR "vitamin B1"[tiab]
  OR "vitamin B-1"[tiab]
  OR folate[tiab]
  OR "folic acid"[tiab]
  OR "vitamin B9"[tiab]
  OR "vitamin B12"[tiab]
  OR cobalamin[tiab]
  OR "vitamin B6"[tiab]
  OR pyridoxine[tiab]
  OR "vitamin D"[tiab]
  OR cholecalciferol[tiab]
  OR "vitamin A"[tiab]
  OR retinol[tiab]
  OR "vitamin E"[tiab]
  OR tocopherol[tiab]
  OR magnesium[tiab]
  OR zinc[tiab]
  OR selenium[tiab]
  OR phosphate[tiab]
  OR phosphorus[tiab]
  OR potassium[tiab]
))
AND
(
  "Cognitive Dysfunction"[Majr]
  OR "Cognition Disorders"[Majr]
  OR "Neurocognitive Disorders"[Majr]
  OR "Neuropsychological Tests"[Mesh]
  OR "cognitive impairment"[tiab]
  OR "cognitive impairments"[tiab]
  OR "cognitive decline"[tiab]
  OR "cognitive dysfunction"[tiab]
  OR "cognitive deterioration"[tiab]
  OR "cognitive deficit"[tiab]
  OR "cognitive deficits"[tiab]
  OR "cognitive performance"[tiab]
  OR "neurocognitive impairment"[tiab]
  OR "neurocognitive dysfunction"[tiab]
  OR "neuropsychological impairment"[tiab]
  OR "neuropsychological performance"[tiab]
  OR "mild cognitive impairment"[tiab]
  OR "executive function"[tiab]
  OR "executive functioning"[tiab]
  OR "processing speed"[tiab]
  OR "working memory"[tiab]
  OR visuospatial[tiab]
)
AND
("Humans"[Mesh] AND ("Adult"[Mesh] OR "Young Adult"[Mesh] OR "Middle Aged"[Mesh] OR "Aged"[Mesh] OR "Aged, 80 and
over"[Mesh]))
```

## Scopus (v4.1)

```
TITLE-ABS-KEY((
 "Alcohol-Related Disorders"
 OR "Alcohol-Induced Disorders, Nervous System"
 OR "Alcoholic Korsakoff Syndrome"
 OR "Alcoholism"
 OR "alcohol use disorder"
 OR "alcohol use disorders"
 OR "alcohol-related disorder"
 OR "alcohol-related disorders"
 OR "alcohol dependence"
 OR "alcohol dependent"
 OR "alcohol abuse"
 OR "alcohol misuse"
 OR "alcohol addiction"
 OR alcoholism
 OR "chronic alcohol use"
 OR "chronic alcohol consumption"
 OR "heavy alcohol use"
 OR "heavy drinking"
 OR "alcohol-related brain damage"
 OR "alcohol related brain damage"
 OR ARBD
))
AND
TITLE-ABS-KEY(((
 "Malnutrition"
 OR "Nutritional Status"
 OR "Nutrition Disorders"
 OR malnutrition
 OR malnourish*
 OR undernutrition
 OR "nutritional deficiency"
 OR "nutritional deficiencies"
 OR "nutrient deficiency"
 OR "nutrient deficiencies"
 OR "nutritional imbalance"
 OR "nutritional imbalances"
 OR "nutritional status"
 OR "nutrient status"
 OR "dietary intake"
 OR "dietary inadequacy"
 OR "nutritional inadequacy"
 OR "poor nutrition"
 OR micronutrient*
 OR "protein-energy malnutrition"
 OR "protein energy malnutrition"
) OR (
 thiamine
 OR thiamin
 OR "vitamin B1"
 OR "vitamin B-1"
 OR folate
 OR "folic acid"
 OR "vitamin B9"
 OR "vitamin B12"
 OR cobalamin
 OR "vitamin B6"
 OR pyridoxine
 OR "vitamin D"
 OR cholecalciferol
 OR "vitamin A"
 OR retinol
 OR "vitamin E"
 OR tocopherol
 OR magnesium
```

```
 OR zinc
 OR selenium
 OR phosphate
 OR phosphorus
 OR potassium
)))
AND
TITLE-ABS-KEY((
 "Cognitive Dysfunction"
 OR "Cognition Disorders"
 OR "Neurocognitive Disorders"
 OR "Neuropsychological Tests"
 OR "cognitive impairment"
 OR "cognitive impairments"
 OR "cognitive decline"
 OR "cognitive dysfunction"
 OR "cognitive deterioration"
 OR "cognitive deficit"
 OR "cognitive deficits"
 OR "cognitive performance"
 OR "neurocognitive impairment"
 OR "neurocognitive dysfunction"
 OR "neuropsychological impairment"
 OR "neuropsychological performance"
 OR "mild cognitive impairment"
 OR "executive function"
 OR "executive functioning"
 OR "processing speed"
 OR "working memory"
 OR visuospatial
))
```

## Web of Science Core Collection (v4.1)

```
TS=(
 "Alcohol-Related Disorders"
 OR "Alcohol-Induced Disorders, Nervous System"
 OR "Alcoholic Korsakoff Syndrome"
 OR "Alcoholism"
 OR "alcohol use disorder"
 OR "alcohol use disorders"
 OR "alcohol-related disorder"
 OR "alcohol-related disorders"
 OR "alcohol dependence"
 OR "alcohol dependent"
 OR "alcohol abuse"
 OR "alcohol misuse"
 OR "alcohol addiction"
 OR alcoholism
 OR "chronic alcohol use"
 OR "chronic alcohol consumption"
 OR "heavy alcohol use"
 OR "heavy drinking"
 OR "alcohol-related brain damage"
 OR "alcohol related brain damage"
 OR ARBD
)
AND
TS=((
 "Malnutrition"
 OR "Nutritional Status"
 OR "Nutrition Disorders"
 OR malnutrition
 OR malnourish*
 OR undernutrition
 OR "nutritional deficiency"
 OR "nutritional deficiencies"
 OR "nutrient deficiency"
 OR "nutrient deficiencies"
 OR "nutritional imbalance"
 OR "nutritional imbalances"
 OR "nutritional status"
 OR "nutrient status"
 OR "dietary intake"
 OR "dietary inadequacy"
 OR "nutritional inadequacy"
 OR "poor nutrition"
 OR micronutrient*
 OR "protein-energy malnutrition"
 OR "protein energy malnutrition"
) OR (
 thiamine
 OR thiamin
 OR "vitamin B1"
 OR "vitamin B-1"
 OR folate
 OR "folic acid"
 OR "vitamin B9"
 OR "vitamin B12"
 OR cobalamin
 OR "vitamin B6"
 OR pyridoxine
 OR "vitamin D"
 OR cholecalciferol
 OR "vitamin A"
 OR retinol
 OR "vitamin E"
 OR tocopherol
 OR magnesium
```

```
 OR zinc
 OR selenium
 OR phosphate
 OR phosphorus
 OR potassium
))
AND
TS=(
 "Cognitive Dysfunction"
 OR "Cognition Disorders"
 OR "Neurocognitive Disorders"
 OR "Neuropsychological Tests"
 OR "cognitive impairment"
 OR "cognitive impairments"
 OR "cognitive decline"
 OR "cognitive dysfunction"
 OR "cognitive deterioration"
 OR "cognitive deficit"
 OR "cognitive deficits"
 OR "cognitive performance"
 OR "neurocognitive impairment"
 OR "neurocognitive dysfunction"
 OR "neuropsychological impairment"
 OR "neuropsychological performance"
 OR "mild cognitive impairment"
 OR "executive function"
 OR "executive functioning"
 OR "processing speed"
 OR "working memory"
 OR visuospatial
)
```

## Europe PMC (v4.1)

```
TITLE_ABS:(
 "Alcohol-Related Disorders"
 OR "Alcohol-Induced Disorders, Nervous System"
 OR "Alcoholic Korsakoff Syndrome"
 OR "Alcoholism"
 OR "alcohol use disorder"
 OR "alcohol use disorders"
 OR "alcohol-related disorder"
 OR "alcohol-related disorders"
 OR "alcohol dependence"
 OR "alcohol dependent"
 OR "alcohol abuse"
 OR "alcohol misuse"
 OR "alcohol addiction"
 OR alcoholism
 OR "chronic alcohol use"
 OR "chronic alcohol consumption"
 OR "heavy alcohol use"
 OR "heavy drinking"
 OR "alcohol-related brain damage"
 OR "alcohol related brain damage"
 OR ARBD
)
AND
TITLE_ABS:((
 "Malnutrition"
 OR "Nutritional Status"
 OR "Nutrition Disorders"
 OR malnutrition
 OR malnourish*
 OR undernutrition
 OR "nutritional deficiency"
 OR "nutritional deficiencies"
 OR "nutrient deficiency"
 OR "nutrient deficiencies"
 OR "nutritional imbalance"
 OR "nutritional imbalances"
 OR "nutritional status"
 OR "nutrient status"
 OR "dietary intake"
 OR "dietary inadequacy"
 OR "nutritional inadequacy"
 OR "poor nutrition"
 OR micronutrient*
 OR "protein-energy malnutrition"
 OR "protein energy malnutrition"
) OR (
 thiamine
 OR thiamin
 OR "vitamin B1"
 OR "vitamin B-1"
 OR folate
 OR "folic acid"
 OR "vitamin B9"
 OR "vitamin B12"
 OR cobalamin
 OR "vitamin B6"
 OR pyridoxine
 OR "vitamin D"
 OR cholecalciferol
 OR "vitamin A"
 OR retinol
 OR "vitamin E"
 OR tocopherol
 OR magnesium
```

```
 OR zinc
 OR selenium
 OR phosphate
 OR phosphorus
 OR potassium
))
AND
TITLE_ABS:(
 "Cognitive Dysfunction"
 OR "Cognition Disorders"
 OR "Neurocognitive Disorders"
 OR "Neuropsychological Tests"
 OR "cognitive impairment"
 OR "cognitive impairments"
 OR "cognitive decline"
 OR "cognitive dysfunction"
 OR "cognitive deterioration"
 OR "cognitive deficit"
 OR "cognitive deficits"
 OR "cognitive performance"
 OR "neurocognitive impairment"
 OR "neurocognitive dysfunction"
 OR "neuropsychological impairment"
 OR "neuropsychological performance"
 OR "mild cognitive impairment"
 OR "executive function"
 OR "executive functioning"
 OR "processing speed"
 OR "working memory"
 OR visuospatial
)
```

## ScienceDirect supplementary discovery query (v3)

```
(
 "Alcohol-Related Disorders"
 OR "Alcohol-Induced Disorders, Nervous System"
 OR "Alcoholic Korsakoff Syndrome"
 OR "Alcoholism"
 OR "alcohol use disorder"
 OR "alcohol use disorders"
 OR "alcohol-related disorder"
 OR "alcohol-related disorders"
 OR "alcohol dependence"
 OR "alcohol dependent"
 OR "alcohol abuse"
 OR "alcohol misuse"
 OR "alcohol addiction"
 OR alcoholism
 OR "chronic alcohol use"
 OR "chronic alcohol consumption"
 OR "heavy alcohol use"
 OR "heavy drinking"
 OR "alcohol-related brain damage"
 OR "alcohol related brain damage"
 OR ARBD
)
AND
((
 "Malnutrition"
 OR "Nutritional Status"
 OR "Nutrition Disorders"
 OR malnutrition
 OR malnourish*
 OR undernutrition
 OR "nutritional deficiency"
 OR "nutritional deficiencies"
 OR "nutrient deficiency"
 OR "nutrient deficiencies"
 OR "nutritional imbalance"
 OR "nutritional imbalances"
 OR "nutritional status"
 OR "nutrient status"
 OR "dietary intake"
 OR "dietary inadequacy"
 OR "nutritional inadequacy"
 OR "poor nutrition"
 OR micronutrient*
 OR "protein-energy malnutrition"
 OR "protein energy malnutrition"
) OR (
 thiamine
 OR thiamin
 OR "vitamin B1"
 OR "vitamin B-1"
 OR folate
 OR "folic acid"
 OR "vitamin B9"
 OR "vitamin B12"
 OR cobalamin
 OR "vitamin B6"
 OR pyridoxine
 OR "vitamin D"
 OR cholecalciferol
 OR "vitamin A"
 OR retinol
 OR "vitamin E"
 OR tocopherol
 OR magnesium
```

```
 OR zinc
 OR selenium
 OR phosphate
 OR phosphorus
 OR potassium
))
AND
(
 "Cognitive Dysfunction"
 OR "Cognition Disorders"
 OR "Neurocognitive Disorders"
 OR "Neuropsychological Tests"
 OR "cognitive impairment"
 OR "cognitive impairments"
 OR "cognitive decline"
 OR "cognitive dysfunction"
 OR "cognitive deterioration"
 OR "cognitive deficit"
 OR "cognitive deficits"
 OR "cognitive performance"
 OR "neurocognitive impairment"
 OR "neurocognitive dysfunction"
 OR "neuropsychological impairment"
 OR "neuropsychological performance"
 OR "mild cognitive impairment"
 OR "executive function"
 OR "executive functioning"
 OR "processing speed"
 OR "working memory"
 OR visuospatial
)
AND
((human* OR patient* OR participant*) AND adult*)
```

## Targeted Vitamin B12 Search Update – 3 October 2026

Following reviewer feedback, we conducted a targeted supplementary search on 3 October 2026 focusing on vitamin B12/cobalamin and cognitive outcomes in adults with alcohol use disorder using PubMed/MEDLINE and Europe PMC. We performed this search separately from the baseline searches conducted on 19 August 2026.

The database-specific search strategies are provided below. The search combined terms related to alcohol use disorder, vitamin B12 and its variants (including B-12, cobalamin, and cyanocobalamin), and cognitive outcomes.

The targeted update yielded 35 deduplicated candidate records, of which one additional eligible full-text study was included in the final synthesis (Reference 4). Consequently, the evidence corpus increased from 44 to 45 sources. The targeted-update records were not included in the baseline search, screening, or retrieval counts.

PubMed / MEDLINE

```
(
 "Alcohol-Related Disorders"
OR "Alcoholism"
   OR "alcohol use disorder"
OR "alcohol dependence"
OR alcoholism
 OR "chronic alcohol use"
)
AND
(
"Vitamin B 12"
 OR "vitamin B12"
OR "vitamin B-12"
```

```
 OR cobalamin
OR cyanocobalamin
)
AND
(
"Cognition Disorders"
OR "Cognitive Dysfunction"
OR cognit*
OR neuropsycholog*
OR memory
OR dementia
OR "brain atrophy"
)
```

Europe PMC

```
TITLE_ABS:(
 "alcohol use disorder"
 OR "alcohol dependence"
OR alcoholism
OR "chronic alcohol use"
)
AND
TITLE_ABS:(
 "vitamin B12"
OR "vitamin B-12"
 OR cobalamin
OR cyanocobalamin
)
AND
TITLE_ABS:(
 cognit*
OR neuropsycholog*
 OR memory
OR dementia
 OR "brain atrophy"
)
```
