# Supplementary material for: Nutritional Exposures and Cognitive Impairment in Alcohol Use Disorder: A Structured Narrative Review of Prioritized Human Evidence
Source: Nutrients. 2026 Oct 27;18(21):3546. doi: 10.3390/nu18213546 (PMC13765479; doi:10.3390/nu18213546)
Supplement: Supplementary file 1 [file nutrients-18-03546-s001.zip › Supplementary Table S1.pdf]

Supplementary Table S1. Study Characteristics and Review-Specific Evidence Appraisal

Levels and grades follow the review-specific rubric supplied with the revision. Effect entries summarize verified local full texts. Contextual sources are not treated as independent effect estimates.

Record / year	Population / setting	Design / timing	Nutritional construct	AUD severity construct	Cognitive outcome class	Effect direction / estimate	Adjustment / confounding	Role / level / grade	Key limitation
AUDNC-0022 (1989)	45 abstinent men with chronic alcoholism	Cross-sectional clinical study; single cross-sectional assessment	Dietary history; folate intake; biochemical nutritional indices; thiamine-pyrophosphate effect	AUD/dependence case definition; no separable severity interaction	domain neuropsychology: Neuropsychological, neurophysiological, and CT measures	NULL_OR_CONTRADICTORY: Moderate folate-intake deficit but no correlation between nutritional and neurological variables	Primarily descriptive/correlation analyses; limited confounder control	DIRECT / IV / C	Cross-sectional study of 45 abstinent men with chronic alcoholism; dietary, biochemical, neuropsychological, neurophysiological, and CT measures. Retain as important contradictory evidence: no nutrition-neurological correlation was found.
AUDNC-0045 (1982)	19 men early in alcohol detoxification	Cross-sectional pilot correlation study; single cross-sectional assessment	Dietary/clinical malnutrition; serum folate; thiamine-related measures	not separately operationalized in extraction	contextual or mixed cognitive outcome: Multitest cognitive battery	MIXED: Folate was the strongest nutritional correlate; thiamine was not associated	Exploratory correlations with extensive multiple testing	DIRECT / IV / C	Cross-sectional study of 19 men early in detoxification. Folic acid was the strongest nutritional correlate of cognitive performance; thiamine was not. Very small sample and extensive correlation testing require caution.
AUDNC-0049 (1995)	Brain tissue from 8 adults with alcoholic cirrhosis plus non-alcoholic controls	Human postmortem case-control study; postmortem	Activities of thiamine-dependent enzymes	not separately operationalized in extraction	imaging/neuropathology: No objective cognition; neuropathological mechanism only	SUPPORTIVE_MECHANISTIC: Reduced transketolase and other thiamine-dependent enzyme activities without overt Wernicke pathology	Postmortem group comparison; terminal liver disease is a major competing explanation	HUMAN_MECHANISTIC_ CONTEXT / IV / C	Postmortem human alcoholic-cirrhosis brain study showing reduced thiamine-dependent enzyme activity. No cognitive testing; use only as human mechanistic context, not direct nutrition-cognition evidence.
AUDNC-0067 (1997)	Alcoholic Wernicke groups with/without Korsakoff amnesia plus alcoholic and non-alcoholic controls	Human postmortem comparative study; postmortem	Neuropathologically defined thiamine-deficiency/Wernicke status	clinical syndrome status; not a general AUD severity scale	domain neuropsychology; imaging/neuropathology: Amnesic phenotype; historical attention assessment; nucleus-basalis cell counts	CONTRADICTORY_MECHANISTIC: Ch4 cell loss was modest and overlapped between amnesic and non-amnesic groups	Small postmortem groups; retrospective clinical classification	HUMAN_MECHANISTIC_ CONTEXT / III / C	Postmortem comparison of alcoholic adults with Wernicke encephalopathy, with and without Korsakoff amnesia, plus alcoholic and non-alcoholic controls. Nucleus-basalis cell loss was modest and did not explain profound memory disorder; use as human neuropathological context, not direct clinical nutrition-cognition evidence.
AUDNC-0078 (2001)	107 adults detoxifying from alcohol before post-randomization losses	Double-blind multidose randomized trial; intervention-period assessment	Five intramuscular thiamine doses over 2 days	not separately operationalized in extraction	global screen; domain neuropsychology: Delayed alternation working-memory task; MMSE; neurological signs	MIXED_SUPPORT: The 200-mg group performed better on delayed alternation; pattern was non-monotonic	Randomization/blinding present; attrition and post-randomization exclusions weaken inference	DIRECT / II / C	Randomized double-blind multidose intramuscular-thiamine study in alcohol-dependent adults. The 200 mg group performed better on delayed alternation, but attrition, post-randomization removals, short treatment, and a non-monotonic dose pattern make the finding preliminary.
AUDNC-0123 (2010)	One 42-year-old adult with reversible alcohol-related dementia	Longitudinal single-case study; baseline plus longitudinal follow-up	No nutritional exposure; thiamine deficiency absent	not separately operationalized in extraction	domain neuropsychology; imaging/neuropathology: Serial neuropsychological tests and FDG-PET over 5 years	SUPPORTIVE_RECOVERY_NO_NUTRITION: Marked cognitive and metabolic recovery with abstinence	Single case; abstinence effect cannot be separated from other recovery factors	PARTIAL_CLINICAL / VI / D	Five-year single-case follow-up of reversible alcohol-related dementia using serial neuropsychological tests and FDG-PET. Cognition and metabolism improved with abstinence, but thiamine deficiency was absent and no nutritional exposure was evaluated; retain only for recovery and differential-diagnosis context.
AUDNC-0134 (2013)	One 56-year-old man with chronic alcohol use	Case report; clinical course/case observation	Low serum niacin; niacin treatment	not separately operationalized in extraction	contextual or mixed cognitive outcome: Clinically described cognitive impairment and encephalopathy	SUPPORTIVE_CASE: Marked global clinical improvement within about 1 week after niacin	No objective cognitive score; co-interventions and diagnostic uncertainty	PARTIAL_CLINICAL / VI / D	Adult alcoholic-pellagra case with low serum niacin and marked clinical improvement after niacin. Cognitive impairment was clinically described rather than objectively quantified, and multiple co-interventions/confounders prevent causal attribution; retain as low-level illustrative evidence.
AUDNC-0138 (2014)	77 patients admitted for alcohol withdrawal and 19 controls	Cross-sectional withdrawal cohort with controls; single cross-sectional assessment	Serum vitamins A, C, and E; nutritional status; eating habits; liver markers	AUD diagnosis plus exposure/history or withdrawal indicator	global screen; imaging/neuropathology: MMSE in a subset; CT brain/cerebellar atrophy	MIXED: Vitamins A/E were lower and related to atrophy indices, but vitamin levels were not related to MMSE	Liver and nutrition analyses performed; subset cognition and cross-sectional design limit inference	DIRECT / IV / C	Observational withdrawal cohort with vitamins A, C, and E, CT brain measures, and MMSE in a subset. Vitamins A/E were lower and related to atrophy measures, but serum vitamins were not related to MMSE performance.
AUDNC-0147 (2014)	One adult with chronic alcohol excess and severe self-neglect	Case report; clinical course/case observation	Measured deficiencies of vitamins A, D, and E; bundled replacement	not separately operationalized in extraction	global screen; clinical mental status/function: MMSE 18/30 and clinical cognitive slowing	MIXED_CASE: Vitamin levels corrected but clinically important cognitive disability persisted	Single case; multifactorial injury and bundled nutritional treatment	DIRECT / VI / D	Adult case of chronic alcohol excess, severe deficiencies of vitamins A, D, and E, and objective cognitive impairment (MMSE 18/30). Multivitamin treatment was bundled and cognition remained impaired, so the contribution of any individual vitamin cannot be isolated.
AUDNC-0158 (2016)	30 adults with AUD and 15 healthy controls	Cross-sectional case-control study; single cross-sectional assessment	Thiamine metabolism; malnutrition; liver fibrosis/function; alcohol exposure	AUD diagnosis plus exposure/history or withdrawal indicator	domain neuropsychology: Executive, visuospatial, verbal episodic-memory, and motor/ataxia domains	SUPPORTIVE_ASSOCIATION: Altered thiamine metabolism predicted episodic-memory impairment; malnutrition predicted a severe profile	Regression models included clinical/biological factors; very small event counts risk overfitting	DIRECT / IV / C	Case-control study of 30 adults with AUD and 15 controls combining alcohol-severity, liver, thiamine, malnutrition, and multidomain neuropsychological measures. Altered thiamine metabolism predicted verbal episodic-memory impairment and malnutrition the severe profile; small samples and model instability require caution.
AUDNC-0170 (2017)	One adult with chronic alcohol use and pellagra-like encephalopathy	Case report; pre-post during treatment/abstinence	Clinical niacin deficiency; nicotinic-acid amide plus multivitamins; normal B1	not separately operationalized in extraction	clinical mental status/function: Clinical mental status and orientation	SUPPORTIVE_CASE: Alertness and orientation improved rapidly after nicotinic-acid amide	No objective cognitive test; simultaneous multivitamins and supportive care	DIRECT / VI / D	Adult alcoholic-pellagra case with progressive cognitive impairment and rapid clinical response to nicotinamide. Retain as low-level, clinically illustrative evidence only.
AUDNC-0174 (2019)	96 adults with chronic alcoholism and 41 controls	Cross-sectional controlled clinical study; single cross-sectional assessment	Whole-blood thiamine; dietary-deficiency Caine criterion; alcohol history	AUD diagnosis plus exposure/history or withdrawal indicator	domain neuropsychology: Six objective cognitive and motor domains	SUPPORTIVE_ASSOCIATION: Low thiamine predicted mnemonic deficits; ataxia predicted non-mnemonic/motor deficits	Regression analyses; polysubstance history and cross-sectional temporality remain limitations	DIRECT / IV / C	Study of 96 adults with chronic alcoholism and 41 controls using Caine nutritional/neurological criteria, whole-blood thiamine, alcohol history, and six cognitive/motor domains. Low thiamine predicted mnemonic deficits, whereas ataxia predicted non-mnemonic and motor deficits; observational design and comorbidity remain important limitations.
AUDNC-0177 (2019)	5,059 thiamine-treated and 5,059 untreated adults with AUD	Nationwide retrospective matched cohort; not applicable or not separately reported	Administrative-record thiamine treatment and cumulative defined daily dose	AUD/dependence case definition; no separable severity interaction	contextual or mixed cognitive outcome: Incident dementia diagnosis	SUPPORTIVE_ASSOCIATION: Adjusted HR about 0.55 (95% CI 0.43-0.69); association present above cumulative DDD 23	Age/sex/index-year matching and multivariable adjustment; indication and residual confounding remain	DIRECT / IV / B	Nationwide claims-based matched AUD cohort (5059 thiamine-treated and 5059 untreated) evaluating incident dementia. Strong association but nonrandomized exposure and residual confounding preclude causal language.
AUDNC-0194 (2020)	61 adults with HIV plus alcohol dependence and 59 controls	Cross-sectional controlled clinical study; single cross-sectional assessment	Dietary-deficiency and other Caine criteria	AUD/dependence case definition; no separable severity interaction	domain neuropsychology: Six objective cognitive and motor domains	SUPPORTIVE_ASSOCIATION: Caine 2+ subgroup showed the most severe attention/working-memory, delayed-memory, and motor deficits	Considered depression, HIV/alcohol variables, AIDS, HCV, and drug history; comorbidity remains substantial	DIRECT / IV / C	Study of 61 adults with comorbid HIV and alcohol dependence and 59 controls. Dietary deficiency and other Caine criteria were assessed alongside objective cognitive/motor domains; participants meeting at least two criteria had the most severe deficits, but HIV, hepatitis C, polysubstance history, and cross-sectional design limit attribution.
AUDNC-0199 (2020)	One 56-year-old man with AUD, malnutrition, and pellagra	Case report; clinical course/case observation	Severely inadequate diet; niacin plus bundled vitamins	AUD/dependence case definition; no separable severity interaction	global screen: MoCA	SUPPORTIVE_CASE: MoCA increased from 20 to 29 during a 5-day admission	Single case; detoxification and multiple supplements administered concurrently	DIRECT / VI / D	Adult AUD pellagra case with severe dietary inadequacy, clinical triad, and

Record / year	Population / setting	Design / timing	Nutritional construct	AUD severity construct	Cognitive outcome class	Effect direction / estimate	Adjustment / confounding	Role / level / grade	Key limitation
									MoCA improvement from 20 to 29 after niacin and bundled vitamin treatment. Retain as low-level direct evidence; single-case design and simultaneous detoxification/supplementation preclude nutrient-specific causality.
AUDNC-0201 (2021)	43 adults with compensated alcoholic cirrhosis; animal component excluded	Prospective human cross-sectional analysis plus animal experiment; single cross-sectional assessment	BMI and dietary calorie/macronutrient pattern	not separately operationalized in extraction	global screen: Korean MMSE	MIXED_SUPPORT: Lower BMI and dietary pattern were associated with selected K-MMSE domains; human causal effect not established	Human analysis is small and observational; liver disease and other confounding remain	DIRECT / IV / C	Human compensated alcoholic-cirrhosis analysis included; mouse diet experiment excluded.
AUDNC-0210 (2023)	100 consecutively admitted adults with alcohol dependence	Prospective single-arm detoxification cohort; pre-post during treatment/abstinence	Blood thiamine and oral thiamine 200 mg/day during 3-week protocol	AUD/dependence case definition; no separable severity interaction	global screen: MoCA and Frontal Assessment Battery	SUPPORTIVE_ASSOCIATION: Baseline thiamine predicted cognition; both thiamine and cognition improved, but their clear association disappeared at discharge	Models considered age, alcohol-toxicity proxies, mood, vitamin D, and liver markers; no untreated control	DIRECT / IV / C	Complete text now verified. Prospective inpatient detoxification cohort with serial thiamine and MoCA; improvement occurred during bundled abstinence, withdrawal care, and oral thiamine, so the thiamine-specific effect is not isolated.
AUDNC-0223 (2025)	86 adults with AUD in inpatient detoxification	Prospective longitudinal cohort analysis; baseline plus longitudinal follow-up	Admission BMI as nutritional proxy	AUD/dependence case definition; no separable severity interaction	domain neuropsychology: Neuropsychological testing at weeks 2 and 6	MOSTLY_NULL: BMI predicted only two speed/visuomotor tasks and not general neurocognitive recovery	Longitudinal modeling but BMI is a crude proxy and extreme-BMI groups were small	DIRECT / IV / C	Longitudinal study of 86 detoxification inpatients with AUD, BMI at admission, and neuropsychological testing at weeks two and six. BMI showed only tentative associations with speed/visuomotor performance and no general relation to recovery; BMI is a crude, non-specific proxy for nutritional deficiency.
AUDNC-0237 (2025)	Long-stay adult AUD clinical population (contextual source)	Clinical-practice guideline/review; not applicable or not separately reported	Dietary deficiency and thiamine-related syndromes	contextual source; no participant-level severity estimand	contextual or mixed cognitive outcome: Cognitive screening, diagnosis, and management recommendations	CONTEXT_ONLY: Provides definitions and management recommendations rather than a new effect estimate	Not primary evidence; guideline-development rigor and grading are limited in the article	GUIDELINE_CONTEXT / VII / C	Clinical-practice guideline addressing definition, assessment, and management of cognitive impairment in long-stay adults with AUD, including dietary deficiency and thiamine-related syndromes. Retain for practice context only, not as primary causal evidence.
AUDNC-0270 (2026)	80 men with alcohol dependence, 42 controls; 41 patients reassessed	Case-control study with longitudinal abstinence subset; baseline plus longitudinal follow-up	No measured nutritional exposure; plasma proBDNF as neuroplasticity marker	AUD/dependence case definition; no separable severity interaction	global screen: MMSE, modified WCST, and verbal fluency	SUPPORTIVE_BIOMARKER_PARTIAL: proBDNF was elevated and related to severity/cognition; changes were weakly related to cognitive recovery	Matched controls and longitudinal subset; male-only sample and attrition limit generalizability	PARTIAL_CLINICAL / IV / C	Case-control and four-week abstinence study of 80 men with alcohol dependence and 42 controls measuring proBDNF, alcohol severity, and objective cognition. It informs neuroplasticity, severity, and recovery but measures no nutritional exposure; biomarker changes were only weakly related to cognitive recovery.
AUDNC-0276 (2025)	7 included studies in adults with AUD	Systematic review; not applicable or not separately reported	Thiamine level/status and supplementation	AUD/dependence case definition; no separable severity interaction	contextual or mixed cognitive outcome: Multiple objective cognitive domains	MIXED_SUPPORT: Most studies suggested lower thiamine related to poorer cognition, including above deficiency cutoffs; evidence remained heterogeneous	Review methods explicit, but few heterogeneous studies and overlap with this review's primary corpus	DIRECT / I / B	Seven-study systematic review retained for evidence mapping and cross-checking of primary studies.
AUDNC-0300 (2022)	30 AUD inpatients and 15 healthy controls	Cross-sectional case-control study; single cross-sectional assessment	Serum TMAO and indoxyl sulfate; prealbumin and nutritional/liver markers	AUD/dependence case definition; no separable severity interaction	global screen: General cognitive assessment	NULL: Neither microbial metabolite was related to AUD severity, withdrawal severity, or cognitive deficit	Exploratory small sample with multiple biological comparisons	DIRECT / IV / C	Study of 30 AUD inpatients and 15 controls measuring microbiota-derived TMAO/indoxyl sulfate, nutritional/liver biomarkers, AUD and withdrawal severity, and general cognition. Neither metabolite related to cognitive deficits or AUD severity; retain this null result and avoid interpreting the metabolites as established nutritional causes.
AUDNC-0301 (2020)	Human imaging plus translational/animal literature	Narrative hypothesis review; not applicable or not separately reported	Thiamine depletion and proposed brain iron overload	contextual source; no participant-level severity estimand	contextual or mixed cognitive outcome: Alcohol-related dementia/cognitive decline (context)	HYPOTHESIS_ONLY: Proposes thiamine-related blood-brain-barrier disruption as a route to brain iron accumulation	Hypothesis not prospectively tested in an adult AUD cohort	HUMAN_MECHANISTIC_ CONTEXT / VII / C	Hypothesis/review proposing that thiamine depletion disrupts blood-brain-barrier integrity and facilitates brain iron overload in AUD. It integrates human imaging with translational and animal findings but does not test the proposed pathway clinically; use only as mechanistic hypothesis context.
AUDNC-0306 (2025)	Commentary on a linked systematic review	Expert commentary; not applicable or not separately reported	Thiamine requirements and supplementation	contextual source; no participant-level severity estimand	contextual or mixed cognitive outcome: Neurocognitive needs (context)	CONTEXT_ONLY: Advocates aligning thiamine treatment research with neurocognitive outcomes	No new data; interpretive dependence on reviewed literature	GUIDELINE_CONTEXT / VII / D	Two-page expert commentary on a linked systematic review. Retain for interpretation and research recommendations only; it is not primary evidence and does not independently establish dose requirements.
AUDNC-0312 (2016)	68 adults with Wernicke-Korsakoff syndrome	Retrospective cohort; not applicable or not separately reported	Clinically diagnosed thiamine deficiency/Wernicke phase; infection markers	clinical syndrome status; not a general AUD severity scale	domain neuropsychology: Later memory and executive neuropsychological tests	SUPPORTIVE_ASSOCIATION: Acute-phase infections were common and associated with lower scores on selected later tests	Retrospective records, substantial missing test data, and illness-severity confounding	DIRECT / IV / C	Retrospective cohort of 68 adults with Wernicke-Korsakoff syndrome linking infection markers during the acute phase with later neuropsychological outcomes. Observational and vulnerable to missing-data and illness-severity confounding.
AUDNC-0316 (2025)	132 alcohol-dependent inpatients during abstinence	Longitudinal genetic-association cohort; baseline plus longitudinal follow-up	MTHFR and VDR polymorphisms; no folate or vitamin-D biomarker	not separately operationalized in extraction	global screen: Repeated MoCA and affective measures	NULL: Tested polymorphisms did not explain cognitive change during abstinence	Genetic pathway proxies do not establish nutrient status; observational follow-up	MECHANISTIC_HUMAN / IV / C	Longitudinal abstinence study of MTHFR/VDR polymorphisms and MoCA in 132 alcohol-dependent inpatients. It measures genetic pathway proxies, not folate or vitamin-D status; the tested variants showed no effect on cognitive change.
AUDNC-0322 (2023)	18 Korsakoff, 47 AUD, and 27 healthy participants	Cross-sectional MRI case-control study; single cross-sectional assessment	No measured nutritional exposure	clinical syndrome status; not a general AUD severity scale	imaging/neuropathology: BEARNI cognitive battery and structural MRI	CONTEXT_PARTIAL: Links BEARNI deficits to structural brain substrates across AUD/KS groups	No nutrient biomarker; group differences and cross-sectional imaging limit causal interpretation	PARTIAL_CLINICAL / IV / C	MRI/BEARNI study in AUD, Korsakoff syndrome, and controls. It supports neurocognitive and structural context, but no nutritional exposure or biomarker was measured; do not label as direct nutrition-cognition evidence.
AUDNC-0335 (2022)	132 alcohol-dependent participants/healthy volunteers across three groups	Cross-sectional case-control study; single cross-sectional assessment	Serum vitamin D	not separately operationalized in extraction	global screen: MoCA and ACE-R	MOSTLY_NULL: Vitamin D differed across groups, but the within-alcohol-dependence cognitive comparison was not significant; all groups were deficient	Cross-sectional, group-composition and residual-confounding concerns	DIRECT / IV / C	Case-control study of 132 participants assessing serum vitamin D with MoCA and ACE-R in alcohol-dependent groups and controls. All groups were vitamin-D deficient and the within-alcohol-dependence comparison was not significant, so the study provides important contradictory/null evidence rather than support for a specific AUD association.
AUDNC-0341 (2023)	50 AUD inpatients undergoing withdrawal treatment	Prospective observational regimen comparison; intervention-period assessment	Blood TPP; intravenous versus oral thiamine regimens	AUD diagnosis plus exposure/history or withdrawal indicator	domain neuropsychology: Repeated cognition including delayed memory	MIXED_SUPPORT: TPP rose by 100.2 nmol/L (95% CI 76.5-123.8); no regimen difference; memory association was secondary/exploratory	No untreated group; abstinence and withdrawal care co-occurred; uncorrected secondary analysis	DIRECT / IV / C	Prospective observational study; delayed-recall association is exploratory and uncorrected.
AUDNC-0348 (2025)	23 adults with moderate-to-severe AUD	Preliminary randomized placebo-controlled trial; intervention-period assessment	N-acetylcysteine 2,400 mg/day versus placebo	moderate-to-severe AUD	contextual or mixed cognitive outcome: Stroop and Trail Making; MRS neurometabolites	NULL: No significant group differences in neurometabolites or cognitive scores after about 19 days	Very small imbalanced groups and short follow-up	DIRECT / II / C	Preliminary randomized placebo-controlled NAC trial in 23 adults with moderate-to-severe AUD using MRS neurometabolites, Stroop, and Trail Making tests. No significant neurometabolic or cognitive benefit was detected; the very small sample and approximately 19-day follow-up limit precision.
AUDNC-0396 (2019)	Adults at risk of Wernicke encephalopathy (contextual source)	Clinical narrative review; not applicable or not separately reported	Thiamine deficiency and treatment route/dose	contextual source; no participant-level severity estimand	clinical mental status/function: Clinical cognitive signs and Korsakoff prevention	CONTEXT_ONLY: Summarizes recognition and prompt treatment; acknowledges dose/route uncertainty	No new comparative data	GUIDELINE_CONTEXT / VII / C	Concise clinical review of Wernicke encephalopathy diagnosis and thiamine treatment. Retain for clinical context; dosage statements remain review-level guidance rather than primary comparative evidence.

Record / year	Population / setting	Design / timing	Nutritional construct	AUD severity construct	Cognitive outcome class	Effect direction / estimate	Adjustment / confounding	Role / level / grade	Key limitation
AUDNC-0409 (2017)	Korsakoff syndrome literature	Critical narrative review; not applicable or not separately reported	Thiamine deficiency versus ethanol neurotoxicity	contextual source; no participant-level severity estimand	domain neuropsychology: Amnesia, executive, behavioral, and social cognition	CONTEXT_ONLY: Argues thiamine deficiency is crucial and evidence for an essential ethanol contribution to WE-to-KS progression is unconvincing	Review-level interpretation; no new effect estimate	HUMAN_MECHANISTIC_CONTEXT / VII / C	Critical review of Korsakoff syndrome covering cognition, thiamine deficiency, ethanol neurotoxicity, neuropathology, diagnosis, and treatment. Retain as etiological and clinical context; its conclusion that thiamine deficiency is crucial is review-level interpretation, not new primary evidence.
AUDNC-0414 (2024)	120 hospitalized patients across alcohol-withdrawal and NAFLD groups	Prospective observational pre-post hospital study; pre-post during treatment/abstinence	B1/B6 plus Cerebrolysin and usual withdrawal care	AUD diagnosis plus exposure/history or withdrawal indicator	global screen: Serial MMSE	SUPPORTIVE_PREPOST_CONFOUNDED: AWS mean MMSE rose from 16.60 to 24.60 over 2 weeks	No untreated AUD comparator; abstinence, withdrawal resolution, vitamins, Cerebrolysin, and practice effects are inseparable	DIRECT / III / C	Romanian hospital study with serial MMSE in patients with alcohol-withdrawal syndrome; mean scores improved after two weeks of abstinence and bundled B1/B6, Cerebrolysin, and usual care. The design cannot separate vitamin effects from abstinence, withdrawal resolution, Cerebrolysin, practice effects, or regression to the mean.
AUDNC-0465 (2012)	Alcohol-related brain-damage clinical context	Expert symposium report; not applicable or not separately reported	Malnutrition and thiamine deficiency	contextual source; no participant-level severity estimand	clinical mental status/function: Clinical cognitive impairment/brain damage	CONTEXT_ONLY: Summarizes mechanisms, prevention, diagnosis, and integrated care	Conference/symposium synthesis without formal evidence grading	GUIDELINE_CONTEXT / VII / D	Medical Council on Alcohol symposium report on alcohol-related brain damage, including thiamine deficiency, diagnosis, prevention, and integrated care. Retain for historical and clinical context only; it is not a primary comparative study or formal evidence-graded guideline.
AUDNC-0478 (2021)	96 AUD detoxification inpatients; MoCA available for 53	Retrospective pilot chart-review study; not applicable or not separately reported	Plasma ascorbic acid; D/B9/B12, albumin and prealbumin	AUD/dependence case definition; no separable severity interaction	global screen: MoCA on days 10-13	SUPPORTIVE_ASSOCIATION: AA deficiency 28.1%; log AA predicted MoCA (beta 1.18, p=.037) after adjustment	Adjusted for multiple factors, but MoCA missing in 45%, selection differed, and temporality is cross-sectional	DIRECT / IV / C	Retrospective association study; no supplementation-effect inference.
AUDNC-0493 (2025)	AUD gut-liver-brain literature	Narrative mechanistic review; not applicable or not separately reported	Gut dysbiosis, permeability, liver pathways, and proposed microbiome interventions	contextual source; no participant-level severity estimand	contextual or mixed cognitive outcome: Cognitive impairment progression (context)	HYPOTHESIS_ONLY: Proposes an interacting microbiome-liver-brain/stress framework for cognitive progression	Human and preclinical evidence are interwoven; therapeutic pathways are hypothesis-generating	HUMAN_MECHANISTIC_CONTEXT / VII / C	Narrative review of the gut microbiome-liver-brain axis, dysbiosis, stress, liver disease, and cognitive impairment in AUD. Retain as hypothesis-generating mechanistic context and clearly separate human observations from extrapolation and proposed therapeutic pathways.
AUDNC-0563 (1995)	Human Wernicke-Korsakoff and animal-model literature	Focused narrative review; not applicable or not separately reported	Alcohol-related thiamine deficiency	contextual source; no participant-level severity estimand	domain neuropsychology: Cognitive and memory dysfunction	CONTEXT_ONLY: Provides historical mechanistic framing of thiamine-related brain injury	Older narrative review with mixed human/animal evidence and no new estimate	HUMAN_MECHANISTIC_CONTEXT / VII / C	Focused review of alcohol-related thiamine deficiency and cognitive/memory dysfunction drawing on human Wernicke-Korsakoff observations and animal models. Retain for historical mechanistic framing, not as an independent primary estimate.
AUDNC-0567 (2022)	393 asymptomatic at-risk and 127 symptomatic alcohol-misuse patients	Two double-blind parallel-group randomized trials; intervention-period assessment	Three parenteral thiamine doses over 3 or 5 days	not separately operationalized in extraction	contextual or mixed cognitive outcome: RUDAS and neurological outcomes	NULL_DOSE_COMPARISON: No clear cognitive/neurological superiority of higher over intermediate/lower doses during examined intervals	Large recruitment but real-world protocol limitations, missingness, and no placebo/no-thiamine arm	DIRECT / II / B	Randomized dose-comparison trials; interpret null result as no observed dose superiority rather than no thiamine effect.
AUDNC-0592 (2025)	30 hospitalized adults aged over 64 with alcohol-risk/AUD screening	Mixed-methods longitudinal descriptive study; baseline plus longitudinal follow-up	24-hour dietary recall and intake relative to recommendations	AUD/dependence case definition; no separable severity interaction	global screen: 6CIT	DESCRIPTIVE_COOCCURRENCE: 53% screened cognitively impaired; median intake for 4 of 5 macronutrients was below recommendations	Small sample, 46.7% attrition, screening-based AUD definition, and no nutrition-cognition model	DIRECT / VI / C	Mixed-methods longitudinal hospital study of 30 adults aged over 64 identified by alcohol screening, with 24-hour dietary recall and 6CIT. Fifty-three percent screened cognitively impaired and most median macronutrient intakes were below recommendations; small sample, high attrition, and a screening-based AUD definition limit inference.
AUDNC-0599 (2026)	Adults with Marchiafava-Bignami disease (context)	Clinical reference chapter; not applicable or not separately reported	Ill-feeding, malnutrition, thiamine and nutritional supplementation	contextual source; no participant-level severity estimand	clinical mental status/function: Clinical dementia/altered consciousness/disconnection features	CONTEXT_ONLY: Summarizes clinical presentation, diagnosis, and treatment rather than new outcomes	Tertiary clinical source; no primary comparative estimate	GUIDELINE_CONTEXT / VII / C	NCBI Bookshelf clinical reference on Marchiafava-Bignami disease in ill-fed adults with AUD, covering presentation, diagnosis, and nutritional treatment. Retain for clinical definitions and management context only, not as primary outcome evidence.
AUDNC-0601 (2024)	21 adults with alcohol-related Marchiafava-Bignami disease	Retrospective case series; clinical course/case observation	Clinical thiamine treatment in 8; thiamine levels generally unavailable	clinical syndrome status; not a general AUD severity scale	clinical mental status/function: Clinical cognitive impairment and Glasgow Outcome Scale	DESCRIPTIVE_CASE_SERIES: Cognitive impairment occurred in 13/21; treatment/outcome data were uncontrolled	No objective cognitive testing, inconsistent nutrient testing, small single-center series	PARTIAL_CLINICAL / VI / D	Retrospective series of 21 adults with alcohol-related Marchiafava-Bignami disease; cognitive impairment was a common clinical symptom and eight received thiamine. Cognition was not objectively tested, thiamine levels were generally unavailable, and treatment was non-comparative; retain as low-level clinical context.
AUDNC-0602 (2024)	160 adults with AUD, 142 with HIV, and 102 controls	Cross-sectional multisample biomarker/MRI study; single cross-sectional assessment	Serum albumin as a non-specific nutrition/liver/inflammation marker	AUD/dependence case definition; no separable severity interaction	domain neuropsychology; imaging/neuropathology: Visual and verbal learning/memory plus other domains; WMH MRI	SUPPORTIVE_ASSOCIATION: Lower albumin independently contributed to periventricular WMH and visual/verbal learning-memory variance	Multivariable adjustment performed; cross-sectional design and albumin non-specificity prevent nutritional causality	DIRECT / IV / C	Human study including 160 adults with AUD, 142 with HIV, and 102 controls. Lower serum albumin was associated with greater periventricular white-matter hyperintensity burden and poorer visual/verbal learning and memory after covariate adjustment; residual confounding and albumin's non-specificity preclude nutritional causality.
AUDNC-0605 (2019)	One 57-year-old man with long-standing heavy alcohol use and acute MBD	Case report; pre-post during treatment/abstinence	Low folate; B1 unmeasured; bundled B-vitamin treatment	not separately operationalized in extraction	clinical mental status/function: Clinical cognitive/mental-status deficits	MIXED_CASE_MINIMAL_RESPONSE: Mental status improved only slightly and other symptoms persisted	No objective cognitive test; multiple co-interventions; inferred rather than measured thiamine deficiency	PARTIAL_CLINICAL / VI / D	Adult alcohol-related Marchiafava-Bignami case with low folate, clinical cognitive deficits, MRI lesions, and bundled B-vitamin treatment. No objective cognitive test was reported, thiamine was not measured, and response was minimal; retain as low-level clinical/mechanistic context.
AUDNC-0607 (2015)	20 hospitalized adults with chronic alcoholism	Non-randomized controlled supplementation pilot; pre-post during treatment/abstinence	Glutathione plus vitamin C with versus without polydatin	AUD/dependence case definition; no separable severity interaction	global screen: MMSE	SUPPORTIVE_PILOT_CONFOUNDED: MMSE improved only in the polydatin-containing group over 2 weeks	Tiny consecutive sample, unclear allocation, abstinence and co-interventions, multiple biochemical outcomes	DIRECT / III / D	Two-week pilot in 20 hospitalized patients comparing antioxidant supplementation with versus without polydatin. MMSE improved only in the polydatin group, but allocation was not described as randomized, groups were tiny, and abstinence/co-interventions confound attribution.
AUDNC-B12-UPDATE (2016)	59 adults with alcohol dependence admitted for major withdrawal symptoms and 24 controls	Observational case-control study; single assessment including blood tests, MMSE and brain CT	Serum vitamin B12, homocysteine, folate and vitamin B6; hepatic-function indices	Alcohol dependence; daily ethanol intake and duration of addiction recorded; no formal nutrition-by-severity interaction	global screen; imaging/neuropathology: MMSE and CT measures of brain atrophy	NULL_OR_CONTRADICTORY: B12 and homocysteine were higher in patients; B12, homocysteine and hepatic indices correlated with brain atrophy, but no independent association with cognitive alterations was established	Multivariable analyses considered liver-function indices and related biomarkers; liver dysfunction remains an important confounder	DIRECT / IV / C	Fernández-Rodríguez et al. (2016), doi:10.1093/alcalc/agw031. Cross-sectional study of 59 alcohol-dependent adults and 24 controls. MMSE was not independently associated with assessed biomarkers. Small sample and confounding by hepatic disease limit causal interpretation.
