# Supplementary material for: Nutritional Exposures and Cognitive Impairment in Alcohol Use Disorder: A Structured Narrative Review of Prioritized Human Evidence
Source: Nutrients. 2026 Oct 27;18(21):3546. doi: 10.3390/nu18213546 (PMC13765479; doi:10.3390/nu18213546)
Supplement: Supplementary file 1 [file nutrients-18-03546-s001.zip › Supplementary Table S2.pdf]

Supplementary Table S2. Record-Level Qualitative Robustness Scenarios and Conclusion Impact

Stable = direction and qualification unchanged; attenuated not reversed = direction retained with lower breadth/confidence; indeterminate = insufficient remaining evidence; reversed = opposite conclusion supported. No quantitative pooling was performed.

The sensitivity analyses were conducted on the original 44-source evidence corpus, before the targeted vitamin B12 search update that increased the final synthesis set to 45 sources.

Panel A. Defined corpus restrictions

ID	Scenario	Rule	Removed n	Remaining n	Removed Record_IDs	Removed themes
SENS-1	Direct primary human evidence only	Retain Evidence_Role=DIRECT and Levels II, III, IV, or VI; remove systematic-review and contextual/partial/mechanistic sources.	19	25	AUDNC-0049; AUDNC-0067; AUDNC-0123; AUDNC-0134; AUDNC-0237; AUDNC-0270; AUDNC-0276; AUDNC-0301; AUDNC-0306; AUDNC-0316; AUDNC-0322; AUDNC-0396; AUDNC-0409; AUDNC-0465; AUDNC-0493; AUDNC-0563; AUDNC-0599; AUDNC-0601; AUDNC-0605	T1=2; T2=1; T4=2; T5=2; T6=12
SENS-2	Exclude case-based/descriptive evidence	Remove every Level VI record.	8	36	AUDNC-0123; AUDNC-0134; AUDNC-0147; AUDNC-0170; AUDNC-0199; AUDNC-0592; AUDNC-0601; AUDNC-0605	T2=4; T3=1; T5=1; T6=2
SENS-3	Remove two high-influence thiamine sources	Remove the nationwide matched cohort and systematic review (AUDNC-0177, AUDNC-0276).	2	42	AUDNC-0177; AUDNC-0276	T1=1; T5=1
SENS-4	Exclude sources remaining preprints at final verification	Remove AUDNC-0605, the only source remaining a preprint at final verification.	1	43	AUDNC-0605	T6=1

Panel B. Impact on central conclusions

Scenario	Conclusion	Impact	Rationale
SENS-1	C1	ATTENUATED_NOT_REVERSED	Direct trials, observational associations, and the matched cohort remain, but review/guideline and mechanistic context is removed.
SENS-1	C2	STABLE	Direct null, mixed, pilot, and case-level findings remain heterogeneous.
SENS-1	C3	STABLE	No retained direct primary study establishes the prespecified modifier/mediation claim.
SENS-1	C4	STABLE	Direct detoxification and supplementation studies still contain the same attribution problem.
SENS-1	C5	ATTENUATED_NOT_REVERSED	Removing contextual mechanisms narrows the framework's evidentiary breadth; it remains explicitly hypothesis-generating.
SENS-2	C1	STABLE	The thiamine conclusion is supported mainly by non-Level-VI sources.
SENS-2	C2	ATTENUATED_NOT_REVERSED	Pellagra and multiple-deficiency evidence loses much of its clinically vivid case support.
SENS-2	C3	STABLE	Removing Level VI evidence does not add or remove a formal interaction test.
SENS-2	C4	STABLE	Controlled and cohort evidence still cannot isolate nutrient effects from withdrawal care consistently.
SENS-2	C5	STABLE	The model remains interpretive and is still supported by non-case mechanistic/contextual evidence.
SENS-3	C1	ATTENUATED_NOT_REVERSED	Prevention and synthesis confidence falls, but biomarker, neuropathology, and dose-comparison evidence remains.
SENS-3	C2	STABLE	The removed sources address thiamine rather than the non-thiamine hierarchy.
SENS-3	C3	STABLE	Neither removed record supplied a formal severity-by-nutrition interaction estimate.
SENS-3	C4	STABLE	The principal attribution limits arise in separate detoxification/intervention studies.
SENS-3	C5	STABLE	The model remains an explicitly unvalidated synthesis.
SENS-4	C1	STABLE	The remaining preprint was not a central source of evidence for thiamine-related cognitive outcomes.
SENS-4	C2	STABLE	Excluding the single preprint removes one low-level severe-syndrome case, while the broader evidence supporting the conclusion remains unchanged.
SENS-4	C3	STABLE	The gap in formal modifier/mediation testing remains.
SENS-4	C4	STABLE	Core longitudinal and intervention evidence is unchanged.
SENS-4	C5	STABLE	Published mechanistic and clinical-context sources still support the framework as a hypothesis.
